# Iterative carotenogenic screens identify combinations of yeast gene deletions that enhance sclareol production

**DOI:** 10.1186/s12934-015-0246-0

**Published:** 2015-04-24

**Authors:** Fotini A Trikka, Alexandros Nikolaidis, Anastasia Athanasakoglou, Aggeliki Andreadelli, Codruta Ignea, Konstantia Kotta, Anagnostis Argiriou, Sotirios C Kampranis, Antonios M Makris

**Affiliations:** Institute of Applied Biosciences/CERTH, P.O. Box 60361, Thermi, 57001 Thessaloniki, Greece; Department of Biochemistry, School of Medicine, University of Crete, P.O. Box 2208, Heraklion, 71003 Greece

**Keywords:** Terpenoids, *Saccharomyces cerevisiae*, Sclareol, Carotenoid, Ergosterol biosynthesis

## Abstract

**Background:**

Terpenoids (isoprenoids) have numerous applications in flavors, fragrances, drugs and biofuels. The number of microbially produced terpenoids is increasing as new biosynthetic pathways are being elucidated. However, efforts to improve terpenoid production in yeast have mostly taken advantage of existing knowledge of the sterol biosynthetic pathway, while many additional factors may affect the output of the engineered system.

**Results:**

Aiming to develop a yeast strain that can support high titers of sclareol, a diterpene of great importance for the perfume industry, we sought to identify gene deletions that improved carotenoid, and thus potentially sclareol, production. Using a carotenogenic screen, the best 100 deletion mutants, out of 4,700 mutant strains, were selected to create a subset for further analysis. To identify combinations of deletions that cooperate to further boost production, iterative carotenogenic screens were applied, and each time the top performing gene deletions were further ranked according to the number of genetic and physical interactions known for each specific gene. The gene selected in each round was deleted and the resulting strain was employed in a new round of selection. This approach led to the development of an EG60 derived haploid strain combining six deletions (*rox1, dos2, yer134c, vba5, ynr063w and ygr259c*) and exhibiting a 40-fold increase in carotenoid and 12-fold increase in sclareol titers, reaching 750 mg/L sclareol in shake flask cultivation.

**Conclusion:**

Using an iterative approach, we identified novel combinations of yeast gene deletions that improve carotenoid and sclareol production titers without compromising strain growth and viability. Most of the identified deletions have not previously been implicated in sterol pathway control. Applying the same approach using a different starting point could yield alternative sets of deletions with similar or improved outcome.

**Electronic supplementary material:**

The online version of this article (doi:10.1186/s12934-015-0246-0) contains supplementary material, which is available to authorized users.

## Background

**Figure 1:**
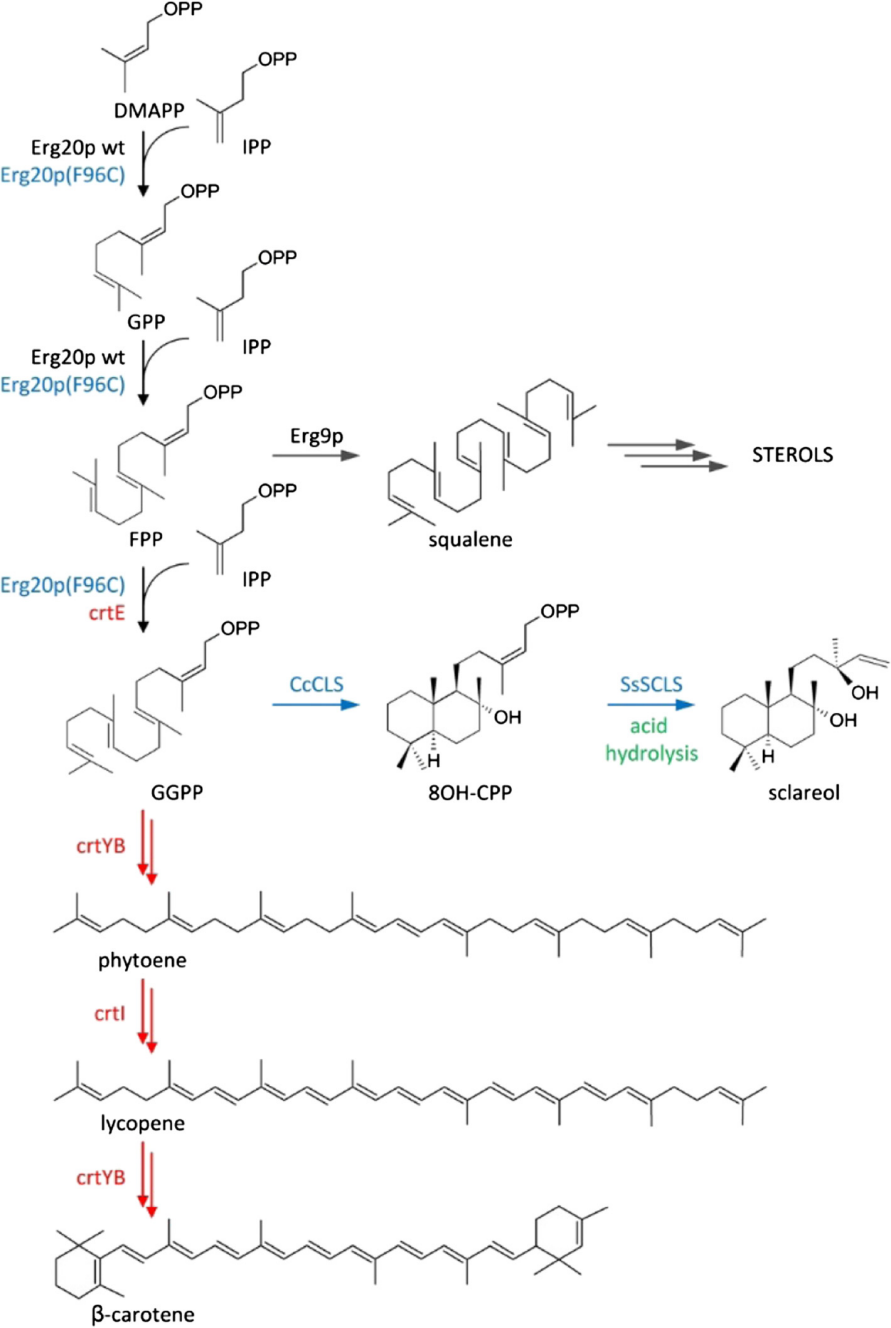
Pathway describing sclareol and carotenoid biosynthesis in yeast. Erg20p catalyzes the formation of C_15_ farnesyl pyrophosphate molecules (FPP) for isoprenoid and sterol biosynthesis. A variant (F96C) enzyme was previously engineered to catalyze geranylgeranyl pyrophosphate synthesis (GGPP). Fusion of *ERG20* (F96C) to the *Cistus creticus* 8-hydroxy copalyl diphosphate synthase (CcCLS) generates 8-OH-CPP, which in turn is converted to sclareol either through spontaneous hydrolysis in the acidified culture medium or enzymatically by the *Salvia sclarea* sclareol synthase (SsSCLS). In carotenoid biosynthesis the inserted carotenogenic pathway cassette of *X. dendrorhous* expresses the the GGPP synthase (encoded by the crtE gene), the bifunctional enzyme phytoene synthase/lycopene cyclase (encoded by the crtYB gene) and phytoene desaturase (encoded by the crtI gene).

Terpenoids (isoprenoids) are an important class of secondary metabolites contributing more than 70,000 compounds to the rich chemical diversity of natural product structures (The Dictionary of Natural Products Online: http://dnp.chemnetbase.com/intro) [1]. Many terpenoids possess pharmaceutical properties and are currently used in clinical practice. Among them are taxol, a diterpene from yew, which has successfully been established as a major antineoplastic agent, and artemisinin, a sesquiterpene lactone, which is an effective antimalarial agent [2-7]. Recently, attention has focused on microbially produced terpenes as biofuels [8-12]. In addition, several terpenes have attracted the interest of the flavour and fragrance industry. Such examples include (+)-nootkatone, an oxidized sesquiterpene extracted from grapefruit [13], santalols, the main components of sandalwood essential oil [14], and sclareol (Figure 1), an industrially important diterpene precursor of a sustainable alternative to ambergris [15,16]. Most of commercially produced sclareol is derived by extraction from cultivated *Salvia sclarea.* The sclareol biosynthetic pathway has recently been elucidated and reconstructed in *E.coli* and *S. cerevisiae* [15,16].

Terpenoids are biosynthesized from two C_5_ precursors, isopentenyl diphosphate (IPP) and dimethylallyl diphosphate (DMAPP) [17]. In yeast and mammals, IPP originates from acetyl-CoA through the intermediate mevalonic acid (MVA). IPP then gives rise to the higher order building blocks, geranyl pyrophosphate (GPP; C_10_), farnesyl pyrophosphate (FPP; C_15_) and geranylgeranyl pyrophosphate (GGPP; C_20_) through the action of prenyltransferases [17]. In yeast, most of the pathway output in the form of FPP is utilized for the biosynthesis of sterols. The terpene hydrocarbon scaffolds are generated by the action of mono-, sesqui-, and diterpene synthases that catalyze multistep reactions using GPP, FPP or GGPP as substrates, respectively. Although *S. cerevisiae* does not produce terpenoids, expression of plant derived terpene synthases in yeast cells revealed that it was possible for the enzymes to utilize the endogenous substrates (GPP, FPP, GGPP) and produce a range of terpenoid compounds [4,18]. The number of terpenoids produced in heterologous systems is continuously growing as more pathways become elucidated and new genes are cloned and characterized. In parallel to the gene discovery effort to identify and characterize enzymes producing chemicals of value, there has been a continuous effort to generate high producing yeast strains. Approaches to improve terpenoid production in yeast have mostly focused on existing knowledge of the sterol biosynthetic pathway with considerable success [4,19,20] (reviewed in [21]). Some key interventions in this direction include a) the deregulation of HMG-CoA reductase (HMGR) by truncation of the regulatory transmembrane domain [22] or point mutations (K6R) in *HMG2* which render the enzyme resistant to ubiquitination [23,24] and b) the suppression of the squalene synthase gene (*ERG9*), which controls the major isoprenoid substrate draining route, that of ergosterol synthesis [4,19,24,25]. However, the magnitude and complexity of genetic interactions identified in yeast cells [26], suggest that the output of a biosynthetic pathway may also be affected by a large number of seemingly unrelated factors. Taking advantage of genetic interaction data, a set of heterozygous gene deletions that increased endogenous hmgp levels and consequently improved sesquiterpene biosynthesis was identified [27].

Quick and inexpensive selection methods have been invaluable tools for strain improvement, as they can dramatically expedite the process and identify novel targets of intervention. Carotenoid biosynthesis has been a goal in itself and a powerful tool for strain improvement [28-32]. Carotenoid formation in yeast utilizes endogenously produced FPP substrate through the activity of a heterologous GGPP synthase, a phytoene synthase, a phytoene desaturase, and a lycopene cyclase (Figure 1) [31,32]. The intensity of the color produced is linked to substrate availability, and allows for rapid screening. Recently Özaydin et al. [29] employed a carotenoid screen on the yeast deletion strain library to identify mutations that can enhance sesquiterpene biosynthesis in yeast. The antioxidant capacity of carotenoids has also been utilized to evolve yeast strains producing high levels of carotenoids using H_2_O_2_ as the prooxidant pressure [30]. A carotenoid screen has also been applied for the selection of yeast Erg20p variants capable of catalyzing all three IPP additions from DMAPP to GGPP, alleviating a major bottleneck for diterpenoid biosynthesis in yeast cells, the low availability of GGPP substrate [33].

In the current study we established and applied a heterozygous deletion screen to identify genes that restrict carotenoid, and potentially diterpene, productivity. We prioritized genes for analysis, on the basis of the lower number of genetic and physical interactions known, to minimize the possibility of the homozygous deletion causing undesirable effects in other cellular pathways. Through an iterative application of this approach, a set of six deletions were identified which led to a 12-fold increase in sclareol titer (750 mg/L) over wild type cells, without obvious effects on yeast robustness and viability.

## Results

### Identification of a working subset of gene deletions that enhance carotenoid production

**Figure 2:**
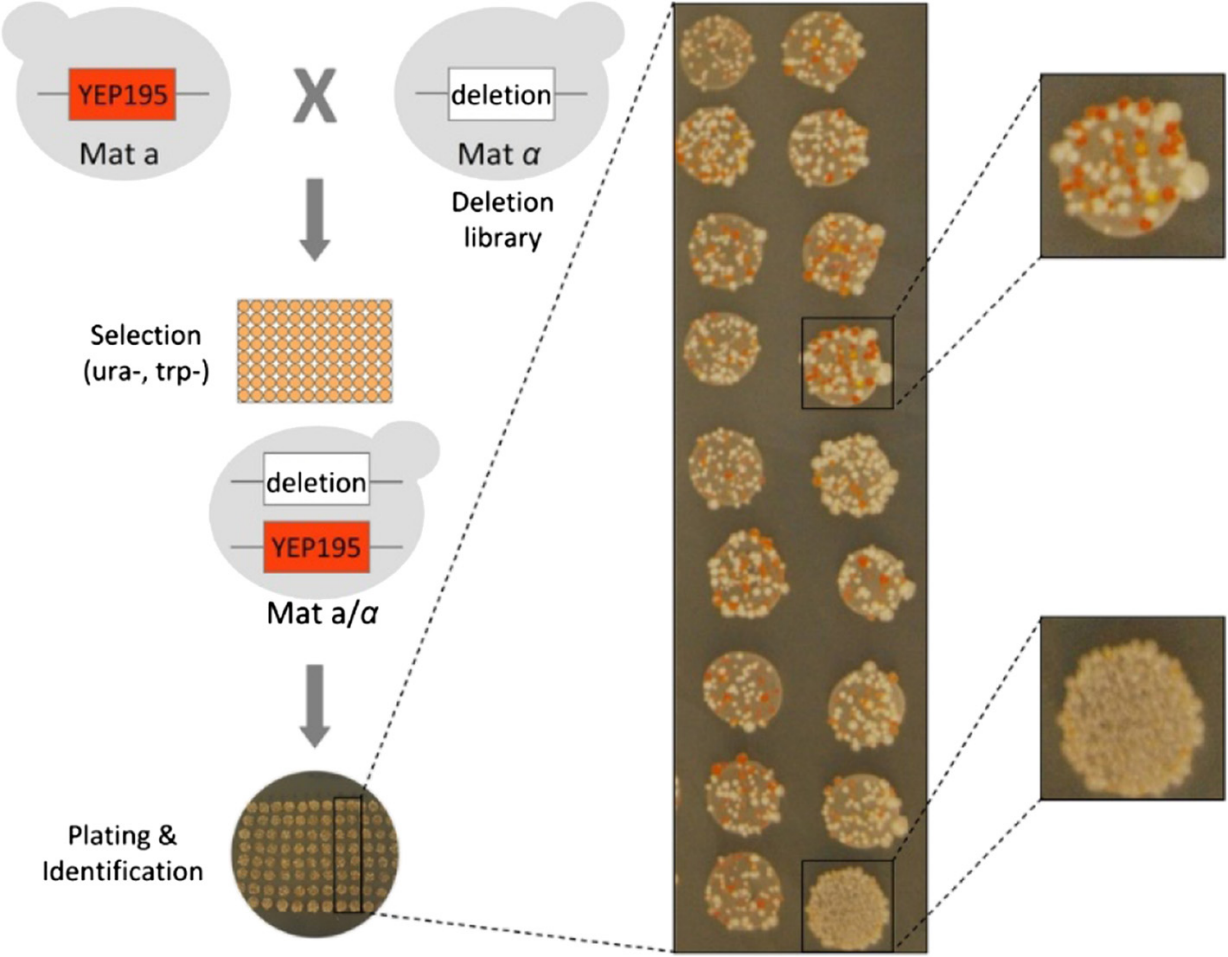
Screening assay to identify heterozygous deletions in yeast which enhance carotenoid biosynthesis. Mat a yeast cells harbouring a chromosomally integrated cassette YEplac195-YB/I/E expressing the GGPP synthase (crtE), the phytoene synthase/lycopene cyclase (crtYB) and the phytoene desaturase (crtI) genes from *Xanthophyllomyces dendrorhous* were mated with the Mat α yeast deletion strain library. Diploid cells were selected and plated. Colonies exhibiting higher percentage of intensely red colonies were selected out for characterization.

A heterozygous gene deletion in *S. cerevisiae* typically results in a 50% decrease in the corresponding protein levels [34]. This allows for the development of a stringent screen that monitors the result of protein depletion in a specific phenotype. By contrast to a haploid or homozygous diploid deletion screen, this approach does not exclude genes whose complete inactivation causes severe growth impediments, and which could eventually be downregulated using more elaborate approaches (e.g. *ERG9* downregulation for isoprenoid production – reviewed in Kampranis and Makris [21]). To identify heterozygous gene deletions in yeast that support increased carotenoid, and consequently diterpene, production, we set out to develop a system to screen the collection of yeast viable deletion strains using a carotenogenic screen. To this end, we developed a haploid yeast strain that expresses the GGPP synthase (crtE), the phytoene synthase/lycopene cyclase (crtYB) and the phytoene desaturase (crtI) genes from *Xanthophyllomyces dendrorhous* and, as a result, produces carotenoids [32]. To maintain a fixed gene copy number and improve reproducibility of carotenoid production, these genes were stably integrated into the genome. This strain was created by transforming EG60 wild type Mat α haploid cells with the linearized vector YEplac195-YB/I/E [32] and selecting transformed cells on glucose CM media lacking uracil. Cells from transformed colonies were subsequently maintained in media without selection. To develop a heterozygous deletion screen, a single colony with moderate and stable carotenoid coloration was grown and crossed to the whole library of 4,700 gene deletion mutants in BY4741 (*MATa*, *his3Δ1, leu2Δ0, met15Δ0, ura3Δ0*) genetic background (Research Genetics) and to BY4741 parental cells as a control. Cells were placed on glucose media lacking uracil and tryptophan, to select for the diploid cells (uracil complementation originates from the YEplac195-YB/I/E vector and tryptophan complementation from the BY4741 genetic background). Visual inspection of the crosses revealed a palette of red color intensities. In some crosses carotenoid formation was abolished completely, most others were similar in coloration to the control, and a small group contained a high percentage of intensely red colored colonies (Figure 2). This group, which consisted of approximately one hundred genes, was selected as a subset for further characterization (Additional file 1: Table S1). Interestingly, eight of the selected genes (*dos2, lcl1, erg3, ipt1, pth1, skn1, sur1 and yor292c*) were also identified in a screen for miconazole resistance [35]. Miconazole interacts with 14α-demethylase, a cytochrome P450 enzyme necessary to convert lanosterol to ergosterol, and the mutants presumably act either by boosting lanosterol production, or by alleviating the effect of ergosterol depletion on yeast membrane function. The selected group was analyzed for enrichment of biological processes by querying the Gene Ontology (GO) experimental dataset for *S. cerevisiae* (http://geneontology.org/) [36]. “Sphingolipid biosynthesis and metabolism” and “cell wall” were top among the enriched terms found, suggesting a role of these deletions on cell membrane function (Table 1).

**Table 1:** **List of Gene Ontology enriched annotated terms for the selected set of genes**

**Term**	**Background frequency**	**Sample frequency**	**Expected**	**+/−**	**P-value**
sphingolipid biosynthetic process (GO:0030148)	22	4	3.313e-01	+	5.855e-03
sphingolipid metabolic process (GO:0006665)	32	4	4.818e-01	+	2.520e-02
carbohydrate derivative metabolic process (GO:1901135)	214	10	3.289e + 00	+	2.760e-02
regulation of transcription factor import into nucleus (GO:0042990)	4	2	6.147e-02	+	2.875e-02
cell wall biogenesis (GO:0042546)	90	6	1.383e + 00	+	4.409e-02
inositolphosphoceramide metabolic process (GO:0006673)	6	2	9.221e-02	+	6.341e-02
peroxisome degradation (GO:0030242)	22	3	3.381e-01	+	7.830e-02
lipid biosynthetic process (GO:0008610)	136	7	2.090e + 00	+	8.220e-02
polysaccharide metabolic process (GO:0005976)	45	4	6.916e-01	+	8.473e-02
membrane lipid biosynthetic process (GO:0046467)	47	4	7.223e-01	+	9.851e-02

**Figure 3:**
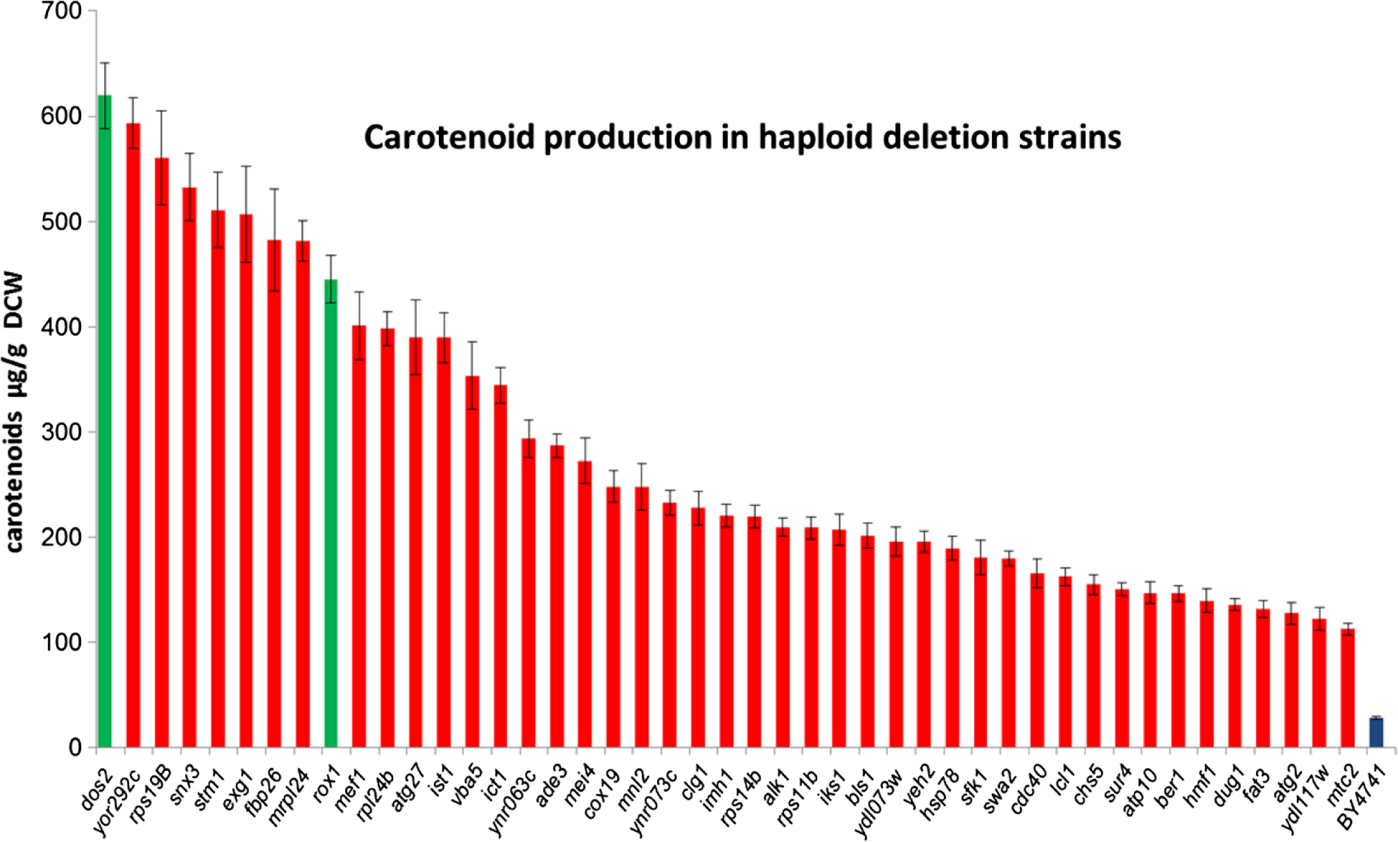
Carotenoid production in selected haploid deletion strains. The selected haploid deletion strains were transformed with YEplac195-YB/I/E plasmid. Cells were grown in 50 ml glucose CM-uracil for 5 days. Carotenoids were extracted from liquid cultures and quantified. In green, *dos2,* the highest producing deletion and *rox1*, a previously identified enhancer of carotenoid biosynthesis. The wild type BY4741 parental is shown in blue color.

To independently validate the contribution of the selected deletions in carotenoid productivity, we also tested the corresponding haploid deletion strains carrying the YEplac195-YB/I/E plasmid episomally. At the time this work was in process, Ӧzaydin and co-workers published results from a similar screen which identified *rox1*, a heme-dependent transcriptional repressor, as an important deletion affecting carotenoid and sesquiterpenoid production [29]. Although the *rox1/ROX1* diploid was not identified as a high carotene producer in our screen, we chose to include *rox1* in our subset. The majority of the deletion strains tested exhibited higher carotenoid yields than control wild type BY4741 cells (Figure 3). The set of the best performing deletions is shown in Figure 3. A subset of 9 deletions that includes *rox1* (*dos2, yor292c, rps19b, snx3, stm1, exg1, fbp26, mrpl24* and *rox1*) stood out with yields >400 μg carotenoids/g dry cell weight (DCW) (Figure 3).

### Combining deletions to increase carotenoid and sclareol productivity

**Figure 4:**
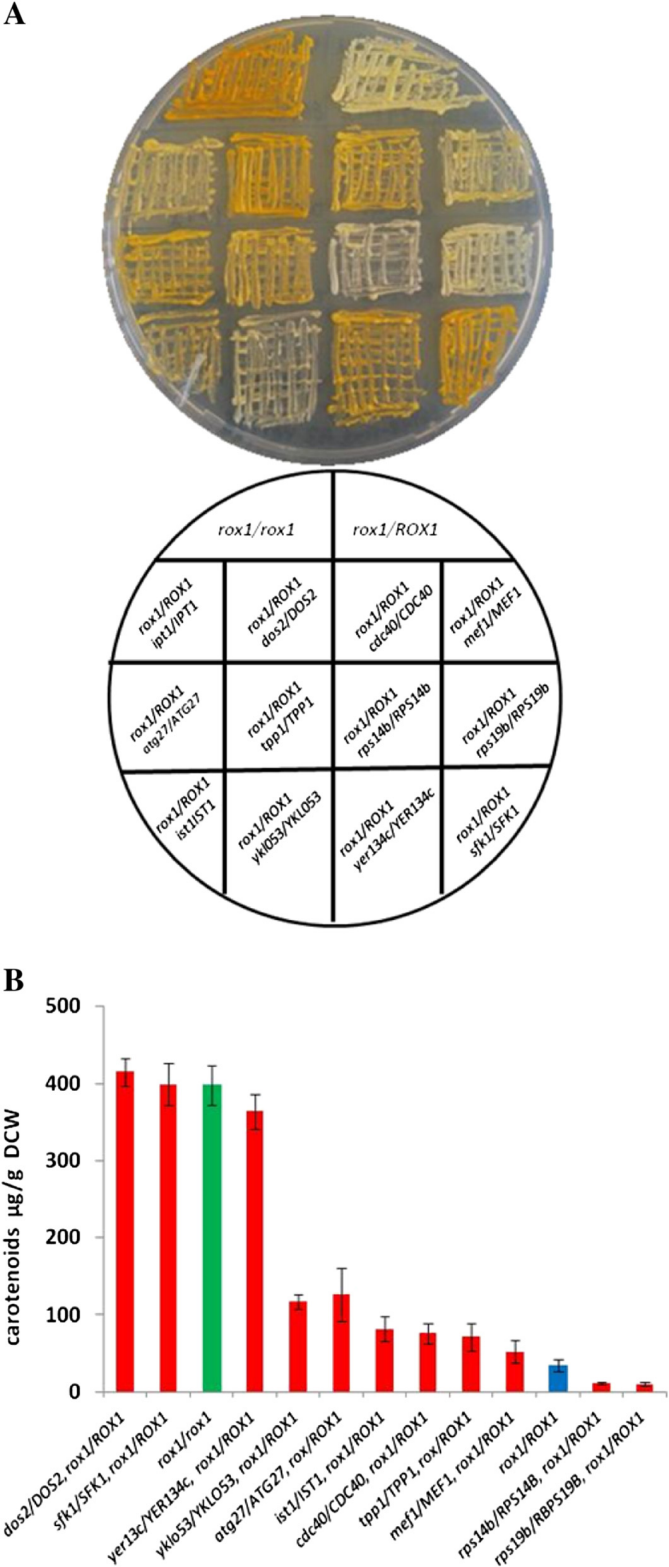
Combined heterozygous deletions can cooperate to enhance carotenoid yields. **(A)** Selected representative double heterozygous deletion strains expressing crtYB/crtI/crtE. L to R. 1st row: *rox1*/*rox1* positive control; *rox1/ROX1* All test strains bellow are heterozygous mutants for *rox1* and the additional deletion tested; 2nd row: *ipt1*; *dos2*; *cdc40*; *mef1*; 3rd row: *atg27*; *tpp1*; *rps14b*; *rps19b*; 4th row: *ist1*; *ykl053*; *yer134c*; *sfk1*, **(Β)** Quantification of extracted carotenoids from three independent cultures of the diploid double heterozygous deletion strains grown in 50 ml glucose CM-U, W. The diploid homozygous *rox1/rox1* is shown in red.

Imbalances in the mevalonate biosynthetic pathway have been shown to cause suppression of terpenoid biosynthesis. The effects of higher levels of *hmgp* depend on the strains’ genetic background [24]. To test whether deletions can be combined to obtain additional productivity increases, we proceeded to generate a *rox1* strain in EG60 background, AM228 (Table 2), and cross it to the selected library subset. After transformation with the YEplac195-YB/I/E plasmid (AM228-1) and mating with the selected subset of strains, a range of color intensities was observed, ranging from very light orange to reddish. As expected, heterozygocity of *rox1/ROX1* produced limited amounts of carotenoids, whereas *rox1/rox1* produced strong color (Figure 4A). Three double heterozygous deletion mutants *rox1/dos2, rox1/sfk1* and *rox1/yer134c* produced very strong coloration and high carotenoid yields (Figure 4A and B). An additional second set which was the most numerous in the visual screen exhibited a lower, but evident, increase in coloration over the control *rox1/ROX1* strain. From the high yielding group of genes, we chose to proceed with *dos2*, as it was consistently picked up in all screens so far. *DOS2* encodes a cytoplasmic protein of unknown function, whose null mutant exhibits increased competitive fitness and resistance to miconazole [35]. To inactivate *DOS2* in the *rox1* haploid strain AM228, we transformed cells with a pUG27 PCR amplified fragment flanked by *DOS2* sequences 5’ and 3’ of the ORF. Integration was validated and the new strain was named AM229 (*rox1, dos2*). Visual inspection of AM229 cells expressing crtYB/I/E showed increased carotenoid production.

**Table 2:** **List of developed yeast strains**

**Strain**	**Genotype**	**Plasmid description**	**Source**
BY4741	*MATa*, *his3*Δ*1, leu2*Δ*0, met15*Δ*0, ura3*Δ*0.*		Research Genetics
EG60	*MATα*, *ura3, trp1, his3.*		Erica Golemis
EG60-2	*MATα*, *ura3, trp1, his3.*	YEplac195-YB/I/E (PTDH3-crtYB-tCYC1, PTDH3-crtI-tCYC1, PTDH3-crtE-tCYC1, URA3, 2 μ)	This study
EG60-3	*MATα*, *trp1, his3* P_TDH3_-crtYB-tCYC1, P_TDH3_-crtI-tCYC1, PTDH3-crtE-tCYC1, *URA3*		This study
EG60-4	*MATα*, *ura3, trp1, his3.*	pUTDH/CLS-ERG20 (F96C) (PTDH3-*CcCLS-ERG20 (F96C)*-tCYC1, 2 μ, *URA3*)	This study
EG60-5	*MATα*, *ura3, trp1, his3.*	pUTDH3/*HMG1*-TM-EYFP, 2 μ PTDH3-*HMG1*-TM-EYFP *URA3*	This study
EG60-6	*MATα*, *ura3, trp1, his3.*	pUTDH3/Sf126-EYFP, 2 μ PTDH3-Sf126-EYFP *URA3*	This study
AM228	*MATα his3, ura3, trp1, rox1* derived from EG60		This study
ΑΜ228-1	*MATα his3, ura3, trp1, rox1* derived from EG60	YEplac195-YB/I/E (PTDH3-crtYB-tCYC1, PTDH3-crtI-tCYC1, PTDH3-crtE-tCYC1, URA3, 2 μ)	This study
ΑΜ228-2	*MATα his3, ura3, trp1, rox1* derived from EG60	pUTDH/CLS-ERG20 (F96C) (P_TDH3_-*CcCLS-ERG20 (F96C)*-tCYC1, 2 μ, *URA3*)	This study
AM229	*MATα his3, ura3, trp1, rox1, dos2* derived from AM228		This study
AM229-1	*MATα his3, ura3, trp1, rox1, dos2* derived from AM228	YEplac195-YB/I/E (PTDH3-crtYB-tCYC1, PTDH3-crtI-tCYC1, PTDH3-crtE-tCYC1, URA3, 2 μ)	This study
AM229-2	*MATα his3, ura3, trp1, rox1, dos2* derived from AM228	pUTDH/CLS-ERG20 (F96C) (P_TDH3_-*CcCLS-ERG20 (F96C)*-tCYC1, 2 μ, *URA3*)	This study
AM230	*MATα his3, ura3, trp1, rox1, dos2, yer134c* derived from AM229		This study
AM230-1	*MATα his3, ura3, trp1, rox1, dos2, yer134c* derived from AM229	YEplac195-YB/I/E (PTDH3-crtYB-tCYC1, PTDH3-crtI-tCYC1, PTDH3-crtE-tCYC1, URA3, 2 μ)	This study
AM230-2	*MATα his3, ura3, trp1, rox1, dos2, yer134c* derived from AM229	pUTDH/CLS-ERG20 (F96C) (P_TDH3_-*CcCLS-ERG20 (F96C)*-tCYC1, 2 μ, *URA3*)	This study
AM231	*MATα his3, ura3, trp1, rox1, dos2, yer134c, vba5* derived from AM230		This study
AM231-1	*MATα his3, ura3, trp1, rox1, dos2, yer134c, vba5* derived from AM230	YEplac195-YB/I/E (PTDH3-crtYB-tCYC1, PTDH3-crtI-tCYC1, PTDH3-crtE-tCYC1, URA3, 2 μ)	This study
AM231-2	*MATα his3, ura3, trp1, rox1, dos2, yer134c, vba5* derived from AM230	pUTDH/CLS-ERG20 (F96C) (P_TDH3_-*CcCLS-ERG20 (F96C)*-tCYC1, 2 μ, *URA3*)	This study
AM233	*MATα his3, ura3, trp1, rox1, dos2, yer134c, vba5, ynr063w* derived from AM231		This study
AM233-1	*MATα his3, ura3, trp1, rox1, dos2, yer134c, vba5, ynr063w* derived from AM231	YEplac195-YB/I/E (PTDH3-crtYB-tCYC1, PTDH3-crtI-tCYC1, PTDH3-crtE-tCYC1, URA3, 2 μ)	This study
AM233-2	*MATα his3, ura3, trp1, rox1, dos2, yer134c, vba5, ynr063w* derived from AM231	pUTDH/CLS-ERG20 (F96C) (P_TDH3_-*CcCLS-ERG20 (F96C)*-tCYC1, 2 μ, *URA3*)	This study
AM238	*MATα his3, ura3, trp1, rox1, dos2, yer134c, vba5, ynr063w, ygr259c* derived from AM233		This study
AM238-1	*MATα his3, ura3, trp1, rox1, dos2, yer134c, vba5, ynr063w, ygr259c* derived from AM233	YEplac195-YB/I/E (PTDH3-crtYB-tCYC1, PTDH3-crtI-tCYC1, PTDH3-crtE-tCYC1, URA3, 2 μ)	This study
AM238-2	*MATα his3, ura3, trp1, rox1, dos2, yer134c, vba5, ynr063w, ygr259c* derived from AM233	pUTDH/CLS-ERG20 (F96C) (PTDH3-*CcCLS-ERG20 (F96C)*-tCYC1, 2 μ, *URA3*)	This study
AM238-3	*MATα his3, ura3, trp1, rox1, dos2, yer134c, vba5, ynr063w, ygr259c* derived from AM233	pUTDH/CLS-ERG20 (F96C) (PTDH3-*CcCLS-ERG20 (F96C)*-tCYC1, 2 μ, *URA3*); pHTDH/CD-*HMG2* (PTDH3-CD-HMG2-tCYC1, 2 μ, *HIS3; p*WTDH/*SCLSmat (*PTDH3*-SCLS-tCYC1, 2 μ TRP1*	This study
AM238-4	*MATα his3, ura3, trp1, rox1, dos2, yer134c, vba5, ynr063w, ygr259c* derived from AM233	pUTDH3/*HMG1*-TM-EYFP, 2 μ PTDH3-*HMG1*-TM-EYFP *URA3*	This study
AM238-5	*MATα his3, ura3, trp1, rox1, dos2, yer134c, vba5, ynr063w, ygr259c* derived from AM233	pUTDH3/Sf126-EYFP, 2 μ PTDH3-Sf126-EYFP *URA3*	This study
AM109-1	*MATa/α*, GALp-(K6R)*HMG2*::HOX2, *ura3, his3, trp1,* PTDH3-*HMG2(K6R)*X2-::*leu2*, PTDH3-*HMG2*(K6R)::HO1, *ERG9/erg9, UBC7/ubc7, ssm4/SSM4, pho86/PHO86,* derived from AM102	pUTDH3/*HMG1*-TM-EYFP, 2 μ PTDH3-*HMG1*-TM-EYFP *URA3*	This study
AM109-2	*MATa/α*, GALp-(K6R)*HMG2*::HOX2, *ura3, his3, trp1,* PTDH3-*HMG2(K6R)*X2-::*leu2*, PTDH3-*HMG2*(K6R)::HO1, *ERG9/erg9, UBC7/ubc7, ssm4/SSM4, pho86/PHO86,* derived from AM102	pUTDH/CLS-ERG20 (F96C) (PTDH3-*CcCLS-ERG20 (F96C)*-tCYC1, 2 μ, *URA3*)	This study

AM229 was crossed to the library subset and tested for color. *YER134c*, identified as a magnesium-dependent acid phosphatase, whose deletion was again among the higher producers. Combined with the low number of genetic (GI = 8) and physical (PI = 6) interactions known, (compiled by Saccharomyces Genome Database http://www.yeastgenome.org/ and curated by Biogrid http://thebiogrid.org/ ), these directed us to target this gene for deletion. The triple deletion strain, AM230 (*rox1, dos2, yer134c*), did not exhibit any growth impediments. In the presence of the YEplac195-YB/I/E plasmid, coloration was increased compared to the parent strain.

**Figure 5:**
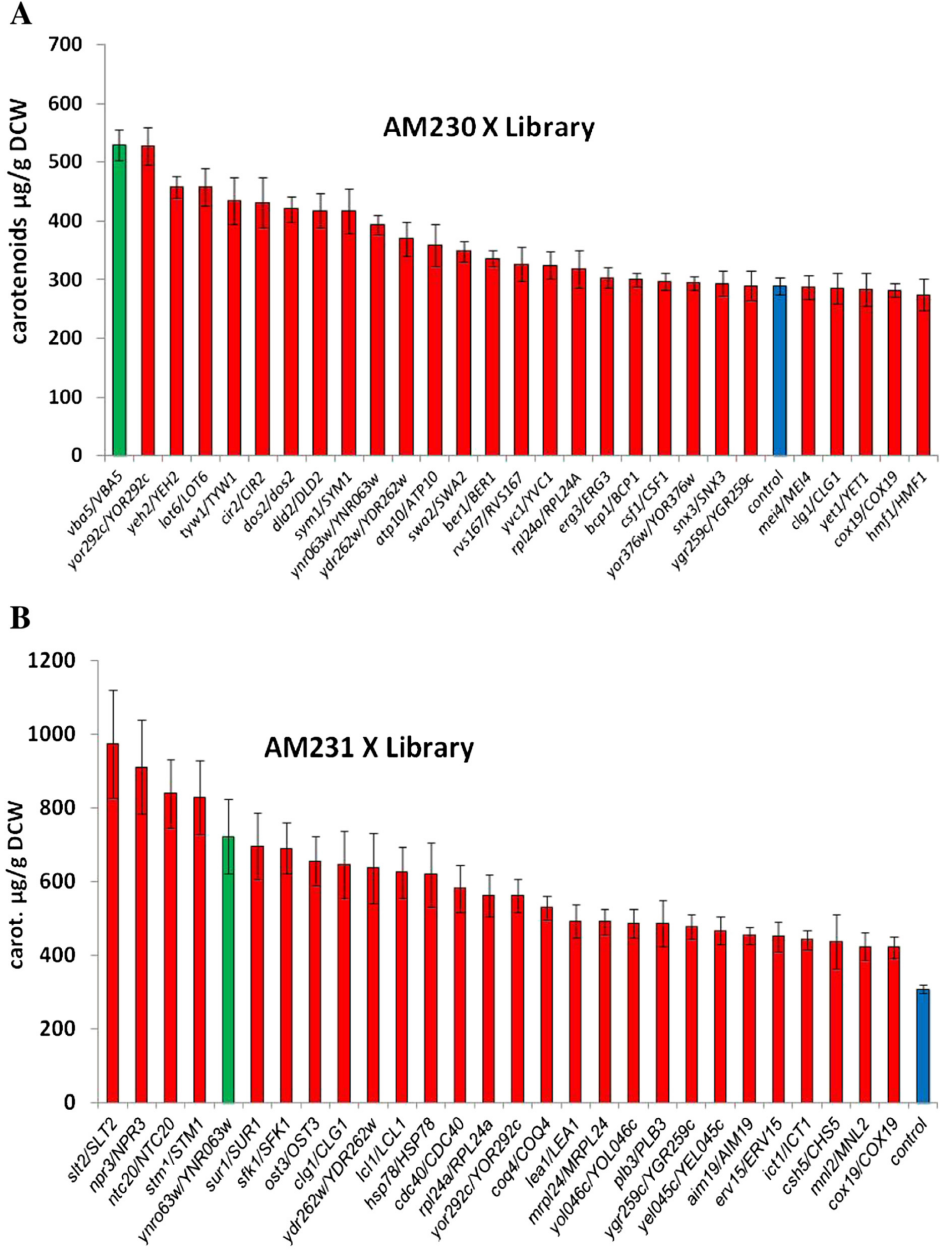
Carotenoid quantification in 4- and 5- heterozygous deletion strains. **(A)** Strain AM230 (*rox1, dos2, yer134c*) expressing crtYB/crtI/crtE was mated to the set of deletion strains, grown and carotenoids were extracted and quantified. The selected *vba5* deletion is shown in green color. Control 3-deletion heterozygous strain is shown in blue; **(B)** Strain AM231 (*rox1, dos2, yer134c, vba5*) expressing crtYB/crtI/crtE was mated to the set of deletion strains as above. In green *ynr063w* selected for additional improvement. Control 4-deletion heterozygous strain is shown in blue.

**Figure 6:**
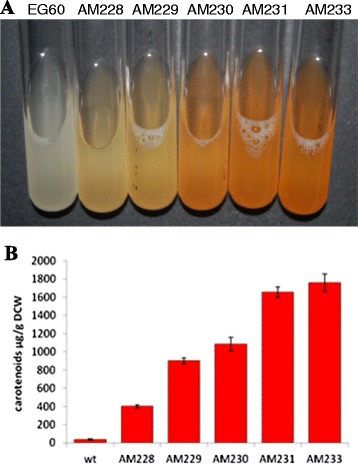
Carotenoid production of improved haploid strains. **(A)** Color formation in yeast cultures due to carotenoids formed in liquid cultures of two independent cultures of AM233 versus control wild type BY4741 cells; **(B)** wild type EG60, AM228 (*rox1*), AM229 (*rox1, dos2*), AM230 (*rox1, dos2, yer134c*) and AM231 (*rox1, dos2, yer134c, vba5*), AM233 (*rox1, dos2, yer134c, vba5, ynr063w*) expressing crtYB/crtI/crtE were grown in independent triplicate cultures and carotenoids were extracted and quantified. Tandem deletions lead to substantial yield improvements.

To identify additional candidates for disruption, the AM230 strain transformed with the YEplac195-YB/I/E plasmid (AM230-1), mated with the selected library subset, and diploid AM230 X Library cells were selected as above. Two heterozygous deletion strains (*vba5/VBA5* and *yor292c/YOR292c*) stood out (Figure 5A). *VBA5* is a vacuolar and plasma membrane amino acid transporter whose deletion increased competitive fitness and replicative lifespan [37]. *YOR292c* is a non-essential vacuolar membrane protein whose deletion led to miconazole resistance [35]. Both have been reported to have a small number of genetic interactions, (GI = 1) and (GI = 2), respectively. We chose to inactivate *vba5* generating strain AM231. The strain was subsequently transformed with the YEplac195-YB/I/E plasmid (AM231-1) and mated once more to the deletion set. Twelve strains yielded significantly higher levels of carotenoids (Figure 5B). From this set, we chose to proceed with *YNR063w*, a putative zinc-cluster protein, being the gene with the fewest interactors (GI = 9) by a long margin from the first four (*slt2* (GI = 554); *npr3* (GI = 91); *ntc20* (GI = 19); *stm1* (GI = 71)), and whose null mutation leads to increased competitive fitness. The *YNR063w* gene was disrupted giving rise to strain AM233 (*rox1, dos2, yer134c, vba5, ynr063w*). As seen in Figure 6A and B, in the process described there is a sequential increase in yields, reaching 1.8 mg carotenoids/g DCW in the case of AM233.

**Figure 7:**
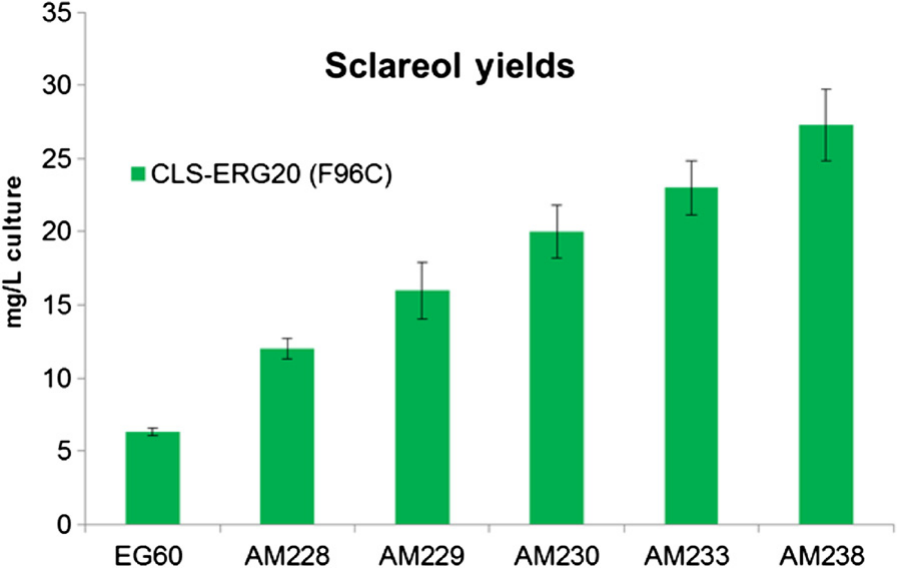
Sclareol titers in engineered yeast strains expressing CLS-ERG20 (F96C). Wild type EG60, AM228, AM229, AM230, AM233 and AM238 cells expressing CLS-ERG20 (F96C) were grown in 5 ml cultures in a roller drum for 24 hours. Subsequently, 500 μl dodecane overlay was added to the media and further incubated for 48 hours. The dodecane phase was analyzed by GC-MS and sclareol was quantified by GC-FID.

An additional round of screening identified *YGR259c*, a small orf which fully overlaps with the larger *TNA1*/YGR260w orf. *TNA1* is a nicotinic acid plasma membrane permease that is found to physically associate with numerous proteins involved in sterol, fatty acid and sphingolipid metabolism [38]. *YGR259c* was deleted, generating strain AM238. The strain was evaluated for carotenoid production but did not show any further increase compared to AM233 (data not shown). At this point it was deemed appropriate to test for sclareol productivity. The base strain EG60 and all the tandem deletion strains were transformed with plasmid expressing a fusion between the *Cistus creticus* 8-hydroxy-copalyl diphosphate synthase (CcCLS) and a mutant form of Erg20p which is capable of producing GGPP [39]. A sequential increase in sclareol yields is observed in each successive sequential strain reaching on average 26 mg/L in AM238 cells compared to 6 mg/L in wt EG60 cells (Figure 7).

### Analysis of the effect of the selected mutations on the sterol biosynthetic pathway

**Figure 8:**
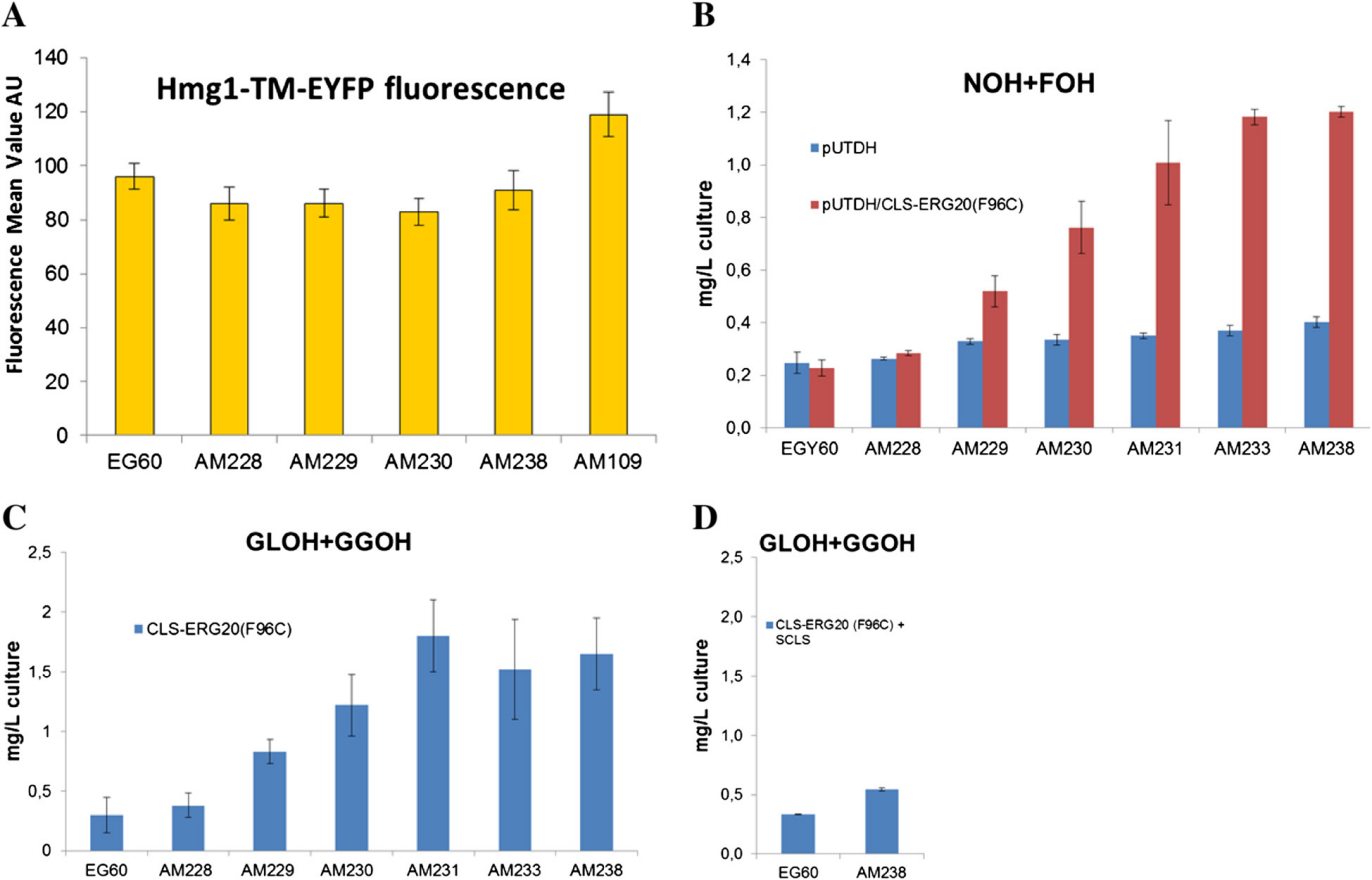
Assessing the state of the mevalonate pathway in engineered strains. **(A)** The transmembrane domain (1–667) of *HMG1* which is regulated by proteolytic degradation was fused to EYFP and expressed by a stable promoter. The mean fluorescence from 50.000 cells per culture in triplicate was measured by flow cytometry. Increased Hmgp stability does not play a role in observed strain improvements; **(B)** Excess FPP and GGPP which is not converted by CLS to 8OH-CPP can be released in the medium and hydrolyzed to farnesol (FOH) and nerolidol (NOH) from FPP, and geranyllinalool (GLOH) and geranylgeraniol (GGOH) from GGPP. These metabolites were quantified in the engineered strains. Cells carrying empty vector show a small increase in NOH, FOH levels at each successive modification. Expression of CLS-*ERG20* (F96C) leads a substantial increase subsequent to the second genetic perturbation (AM229); **(C)** GLOH + GGOH are increasing after the second perturbation; **(D)** co-expression of CLS-*ERG20* (F96C) with SCLS reduced the levels of released GLOH + GGOH, as substrate is efficiently converted to sclareol.

In a previous effort to develop optimal strains for sesquiterpene production by disrupting single alleles of *HMG1* and *HMG2* genetic interactors, the developed strains exhibited increased stability of Hmg1p, suggesting a function for the selected deletions [27]. To assess whether the strains developed here also exhibit improved Hmg1p stability, we transformed EG60, AM228, AM229, AM230, AM238, and a positive control, AM109 [27], strains with plasmid pUTDH/HMG1-TM-EYFP. This plasmid expresses a fusion of the transmembrane domain (TM) of Hmg1p to EYFP [27]. Fifty thousand cells from each culture grown in triplicate were measured by flow cytometry and the mean fluorescence intensity was estimated. Hmg1p-TM-EYFP fluorescence initially decreased somewhat after the *rox1* deletion but recovered to wild type levels in AM238 cells. In contrast, fluorescence increased substantially in AM109 cells (Figure 8A), suggesting that the carotenoid/diterpene and sclareol increase observed in the engineered strains is not due to Hmgp stabilization.

**Figure 9:**
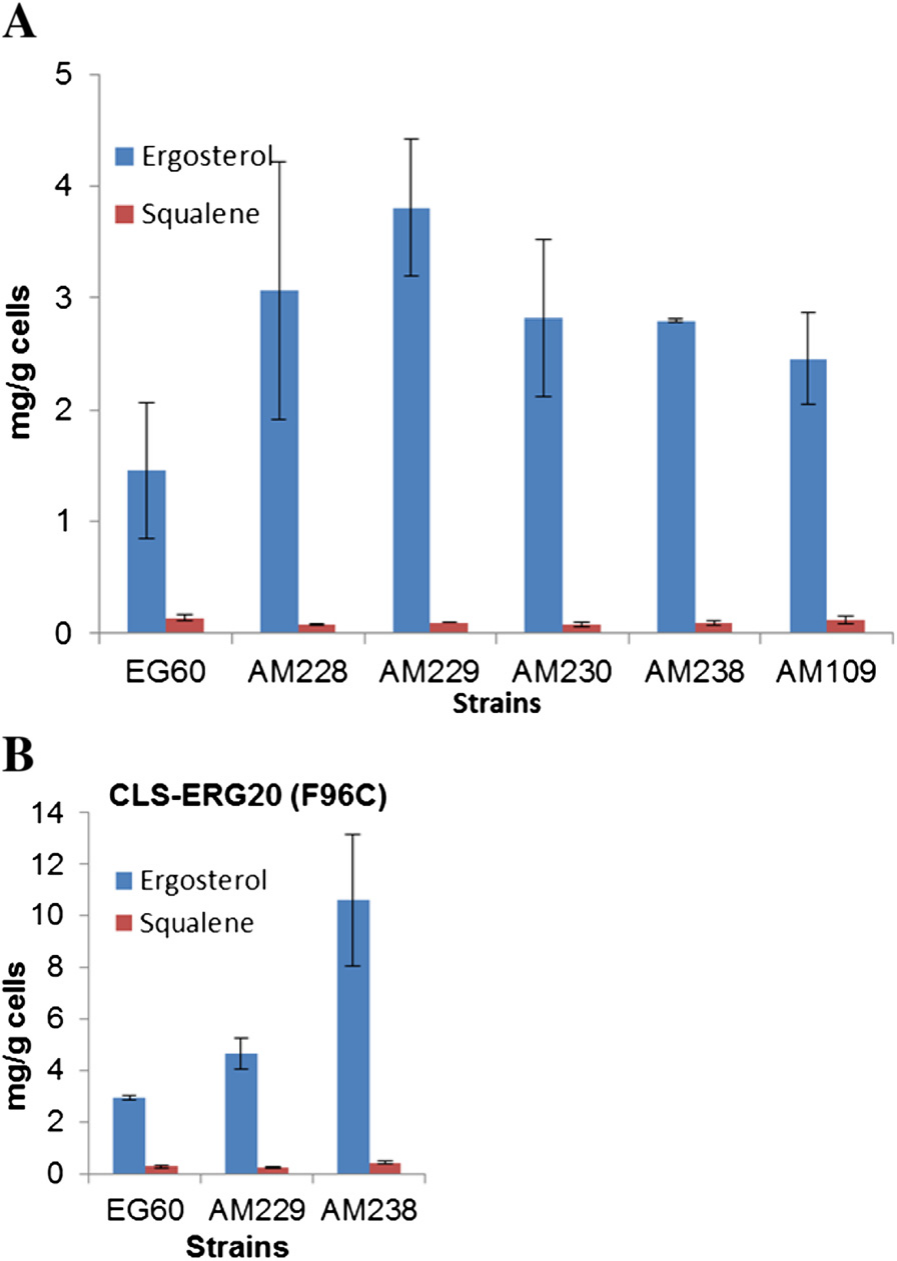
Increased ergosterol yield in engineered strains. **(A)** Ergosterol and squalene quantification in strains EG60, AM228, AM229, AM230, AM238 and AM109 cells; **(B)** Ergosterol and squalene quantification in EG60, AM229, AM238 cells overexpressing CLS-*ERG20* (F96C).

Excess FPP and GGPP that is not converted by CLS to 8OH-CPP and subsequently to sclareol, can be released to the medium and hydrolyzed to farnesol (FOH) and nerolidol (NOH) from FPP, and geranyllinalool (GLOH) and geranylgeraniol (GGOH) from GGPP [39]. These metabolites were quantified in all engineered strains. Strains carrying empty vector showed a small increase in NOH + FOH levels in each successive modification (Figure 8B). Expression of CLS-ERG20 (F96C) fusion led to a substantial increase in NOH + FOH levels subsequent to the second deletion (AM229 *rox1 dos2*, AM230 *rox1 dos2 yer134c*, AM231 *rox1 dos2 yer134c vba5*, AM233 *rox1 dos2 yer134c vba5 ynr063w*, AM238 *rox1 dos2 yer134c vba5 ynr063w ygr259c* ) (Figure 8B). The released sesquiterpenols in AM238 cells expressing CLS-*ERG20* (F96C) were at 4-fold higher levels than the corresponding wild type cells. No GLOH + GGOH was detected in all strains carrying empty vector (not shown). Among the strains expressing CLS-*ERG20* (F96C) increasing levels of GLOH + GGOH was subsequent to the second deletion (Figure 9C). Sclareol synthase (SCLS) a class I diterpene synthase (diTPS), previously isolated from *Salvia sclarea*, converts 8OH-CPP to sclareol and manool [15]. Co-expression of SCLS in wild type EG60 and AM238 cells reduced levels of released GLOH + GGOH, indicating that the enzymatic conversion can be more efficient than spontaneous hydrolysis (Figure 9D). NOH + FOH levels were not reduced to a great extent (data not shown).

In the sterol biosynthetic pathway, FPP gives rise to squalene, which, in turn, is converted through a series of steps to ergosterol. The amounts of ergosterol and squalene produced by strains EG60, AM228, AM229, AM230, AM238 and AM109 were quantified. Deletion of *rox1* in strain AM228 led to ergosterol increase. This is not surprising a *ROX1* is a known suppressor of the ergosterol biosynthetic pathway [40]. This increase is maintained in the successive engineered strains (AM229, AM230, AM238), suggesting a plateau in ergosterol levels (Figure 9A). Upon overexpression of the CLS-*ERG20* (F96C) fusion in EG60, AM229 and AM238 cells, ergosterol levels in the latter two increased substantially, reaching an average of 10.6 mg/g ergosterol for AM238 cells (Figure 9B).

**Figure 10:**
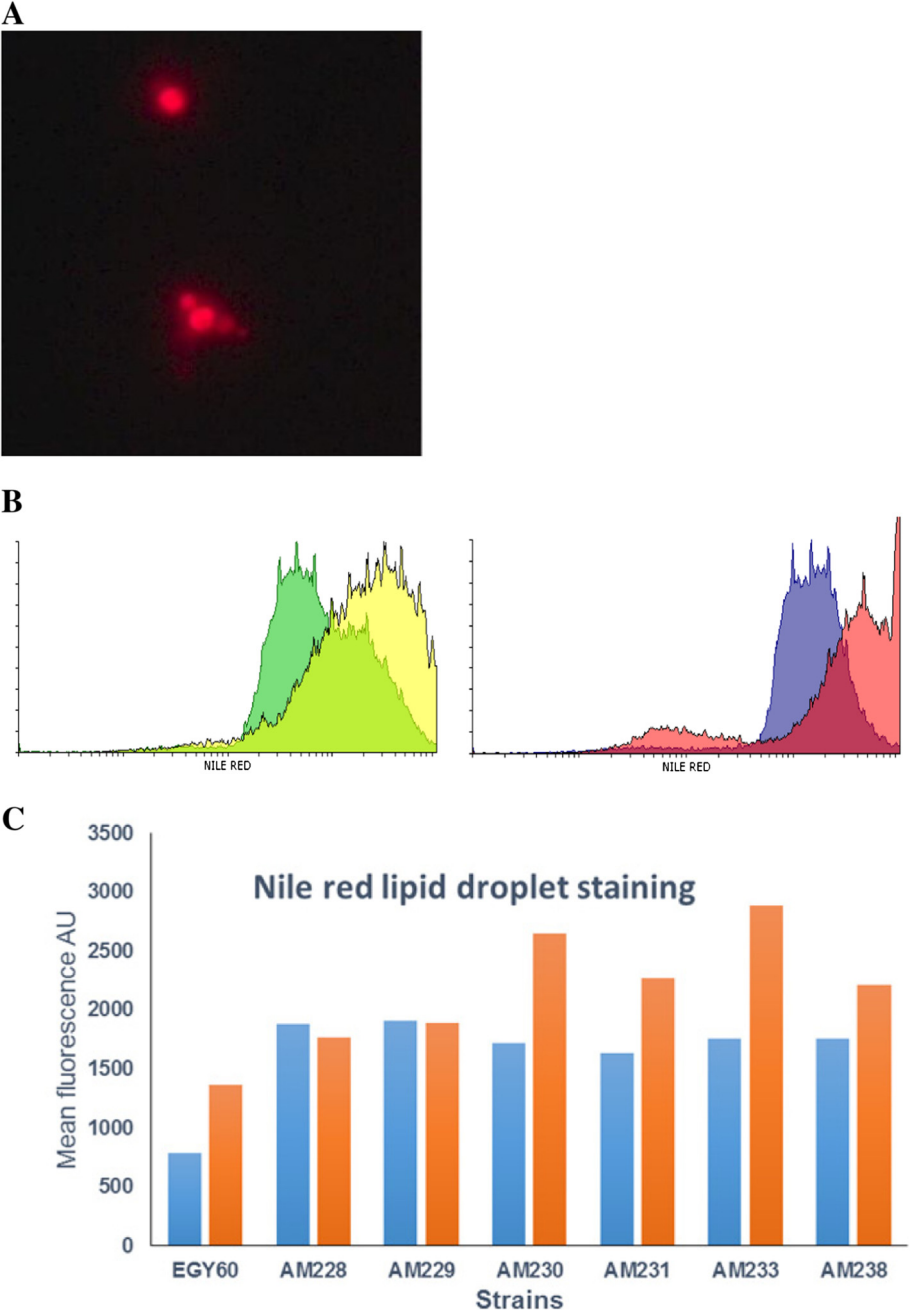
Lipid droplet quantification in the engineered strains. **(A)** Lipid droplet accumulation in 5 days old culture of yeast visualized by Nile red staining; **(B)** Overlays of flow cytometry analysis in EG60 cells (left) with empty vector (green) or expressing CLS-*ERG20* (F96C) (yellow), and AM238 cells (right) with empty vector (blue) or expressing CLS-*ERG20* (F96C) (red); **(C)** Mean fluorescence values of strains carrying empty vector (blue) or expressing CLS-*ERG20* (F96C) (orange). 50.000 cells were counted from each strain.

Excess lipids are stored intracellularly as lipid droplets, ubiquitous organelles composed of a neutral lipid core surrounded by a phospholipid monolayer. In yeast, lipid particles consist of triacylglycerols (TGs) and steryl esters (SE) roughly in equal amounts. To assess whether the observed changes in ergosterol levels are reflected in the stored lipid contents, cells were stained with Nile Red, a lipophilic dye accumulating in neutral lipid deposits. Strains EG60, AM228, AM229, AM230, AM231, AM233, AM238 carrying either empty vector or expressing CLS-*ERG20* (F96C) were grown to saturation over a 5 day period and were stained with Nile Red. Distinct intracellular structures are evident by fluorescent microscopy (Figure 10A). Fluorescence intensity was quantified in 50.000 cells from each strain by flow cytometry. Plot overlays of cell counts to fluorescence intensity were generated (Figure 10B) and the mean fluorescence intensities were calculated (Figure 10C). Deletion of *rox1* in AM228 cells resulted in fluorescence increase which remained steady at subsequent perturbations. Expression of CLS-*ERG20* (F96C) led to increased fluorescence subsequent to the third deletion (strains AM230, AM231, AM233, and AM238). The Nile Red results agree with the observed ergosterol increases in AM238 cells expressing CLS-*ERG20* (F96C) (Figure 9B) and the NOH + FOH increased yields (Figure 8B). Overall, it appears that the initial genetic perturbations (AM228, AM229, AM230, and AM231) lead to increases in the mevalonate pathway biosynthesis, whereas for the subsequent deletions (AM233, AM238) improvements in yields are not due to increased substrate production.

**Figure 11:**
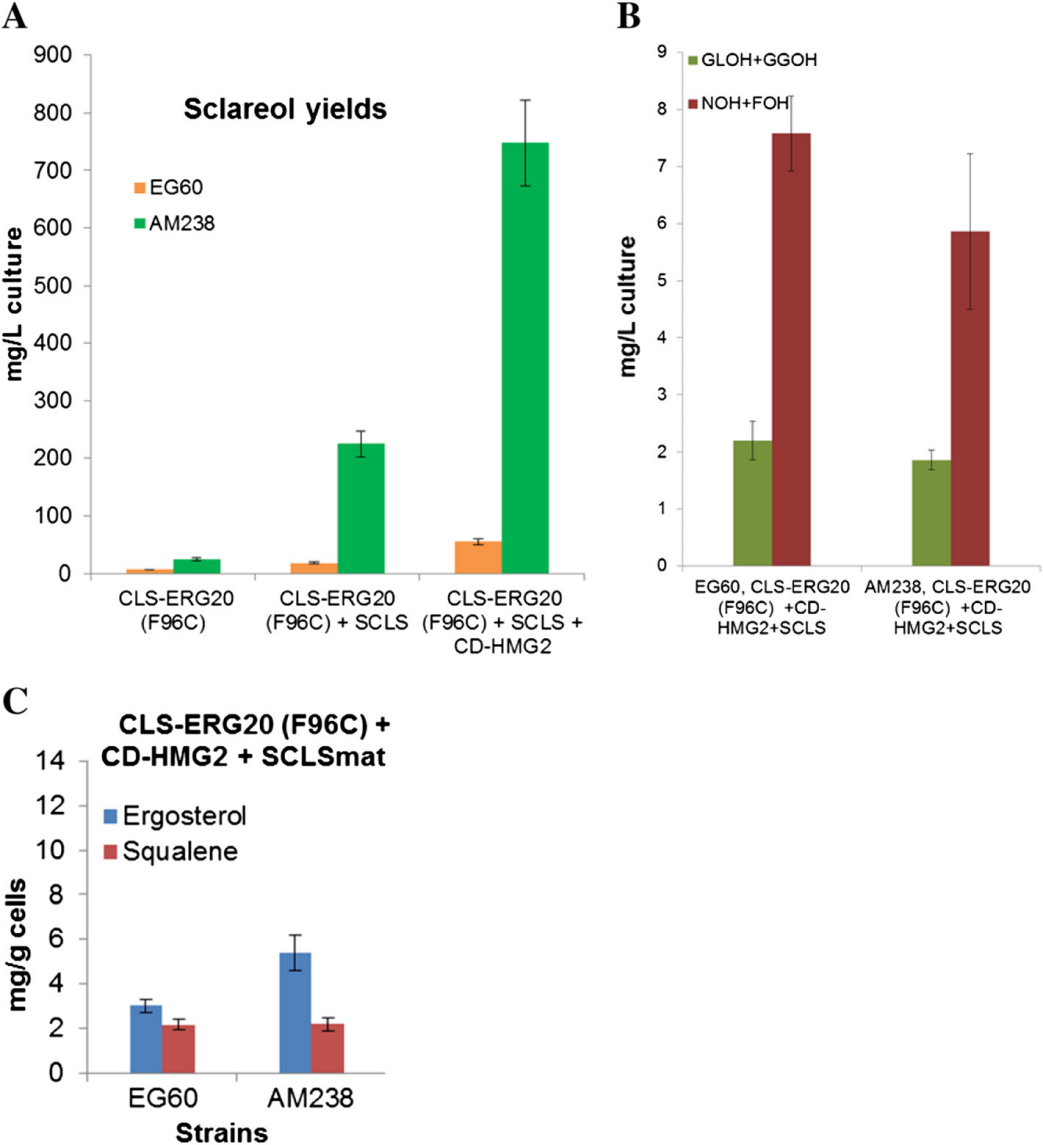
Maximizing sclareol titers in AM238 cells. **(A)** EG60 and AM238 cells expressing CLS-*ERG20* (F96C) only, CLS-*ERG20* (F96C) with sclareol synthase SCLS, and CLS-*ERG20* (F96C), SCLS with CD-*HMG2*. The engineered iterative perturbations generated a strain producing 12-fold higher sclareol levels than wild type cells; **(B)** Released NOH + FOH and GLOH + GGOH in EG60 and AM238 cells expressing the three plasmids. A reduction of released terpenols is seen in AM238 cells indicating a more efficient substrate conversion to sclareol; **(C)** Ergosterol and squalene levels in EG60 and AM238 cells expressing the three plasmids. Ergosterol levels are reduced by half in AM238 cells co-expressing SCLS and CD-*HMG2*, compared to CLS-*ERG20* (F96C) only. Squalene levels are significantly increased by CD-*HMG2* expression.

Taking into consideration our previous results, it appears that the sclareol conversion process could be further optimized by co-expressing, the mature form of sclareol synthase from *Salvia sclarea* (SCLS)*,* and the catalytic domain of *HMG2* (CD-*HMG2*) which encodes for a constitutively active 3-hydroxy-3-methyl-glutaryl-CoA reductase, a key enzyme in the control of the mevalonate pathway. Whereas wild type EG60 cells co-expressing the above three genes produced only 62 mg/L, AM238 cells co-expressing the same genes reached titres of 743 mg/L, a 12-fold increase (Figure 11A).

Wild type EG60 and AM238 strains co-expressing CLS-*ERG20* (F96C), CD-*HMG2*, and SCLS expectedly released substantially higher NOH + FOH due to the presence of the CD-*HMG2* (7.58 mg/L for EG60 and 5.86 mg/L for AM238 strain). Released GLOH + GGOH averaged to 2.2 mg/L for EG60 and 1.86 mg/L for AM238, indicating a higher proportion of substrates being converted to final products in AM238 cells (Figure 11B). Ergosterol levels were reduced by half in AM238 cells, averaging 5.4 mg/g compared to AM238 cells expressing CLS- *ERG20* (F96C) only (Figure 9B). At the same time squalene levels increased substantially in both strains, which is attributed to the effects of CD-*HMG2* expression. Co-expression of SCLS and CD-*HMG2* led to substantial improvements in substrate availability and utilization and targeting *ERG9* could further reduce FPP flow towards sterol synthesis and lead to additional sclareol yields.

## Discussion

**Figure 12:**
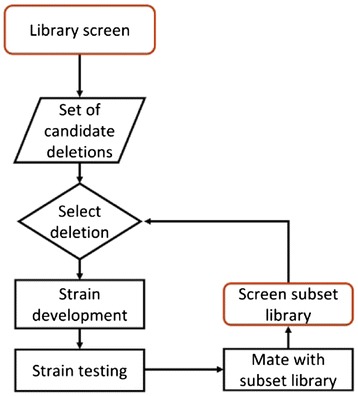
Outline of iterative approach to identify novel combinations of gene deletions improving carotenoid and sclareol production.

Aiming to develop a productive yeast platform for sclareol production, we undertook a screen of deletions strains suitable for diterpene biosynthesis. The carotenoid screen has been successfully used in the past for discovering yeast deletions which would improve sesquiterpenoid production [29]. We developed and applied an alternative version of the screen which would enable us to identify haploinsufficiencies which enhance carotenoid productivity (Figure 12). To avoid the issue of plasmid instability in our screen which results in white colonies the YB/I/E cluster was integrated into the genome. However, the heterogeneity of carotenogenesis remained despite cluster integration. An alternative explanation could be that GGPP formation causes feedback inhibition to components of the ergosterol biosynthetic pathway [41]. This screen enabled the selection of a set of approximately 100 candidate strains exhibiting improved carotenoid productivity. The set of identified gene deletions did not overlap with the corresponding ones from the Ozäydin et al. screen. Deletion in *ERG3* identified in the current screen could be functionally equivalent to *ERG24* deletion previously found, as both genes code for interacting enzymes of the ergosterol biosynthetic pathway. The alternative carotenogenic screen applied here requires a dominant effect on a heterozygous background, as can be seen in the case of *ROX1* deletion (Figure 4. Most strains also exhibited improvement in sclareol production. Iteration of the carotenonogenic screen incorporating the previously selected perturbations allowed stepwise improvement in carotenoid yields. The successive compilations of deletions during repetitions of the carotenoid screen, made evident that the previous deletion genetic background altered the performance of the newly introduced ones. Deletions which perform well in a given genetic background subsequently lose their significance, while others with an initial mild effect later show increased impact. This is not surprising, as deletions affecting the same, or other cooperating or competing pathways can reach different final output readout. Imbalances caused by uneven effects on the biosynthetic pathway can also lead to decreased productivity. The iterative screen approach allows the identification of perturbations for which no previous knowledge of their involvement exists, and the compilation of compatible perturbations improving the final output.

In our carotenoid screen, eight of the deletions in the set had been re-identified in a screen for resistance to miconazole. The main mode of azole function is the inhibition of ergosterol biosynthesis. Several of the miconazole resistant deletion resistant mutants (*IPT1, SKN1, SUR1, ERG3*) affected sphingolipid and ergosterol biosynthesis, molecules enriched in lipid rafts [35]. Membrane rafts are thought to compartmentalize the plasma membrane and play an important role in cell signaling. It is plausible that signaling from lipid rafts may relay perceived membrane sterol levels to the regulatory machinery. Interestingly, *rox1* deletion which was identified in a previous carotenoid screen as inducer of carotenoid biosynthesis, also exhibited decreased susceptibility to azole compounds. For miconazole specifically, the 50% Inhibitory Concentration, IC_50,_ increased by 2.5-fold on average [42]. *ROX1* deletion was found to result in increased expression of most ERG biosynthetic genes. In models of ergosterol pathway transcriptional control *ROX1* together with *MOT3* is thought to repress *ECM22* upon osmotic stress and exert a direct effect by binding on several ERG promoters [40]. Still, knowledge is lacking as to the signaling pathways regulating ergosterol homeostasis. Presumably, some of the newly identified deletions may play roles in such complex regulatory processes. Our screening setup enabled us to match the line of deletions which can act cooperatively and incrementally increase the availability of substrate. *DOS2* deletion was judged to be a suitable combination with *ROX1* inactivation. The *DOS2* gene (YDR068w) had also been identified in the miconazole screen; its haploid deletion exhibited the highest levels of carotenoid formation, and as heterozygous deletion with *rox1*, it exerted a similar effect. Desiring to maintain the option of using the deletions in a haploid or diploid heterozygous genetic background, we selected the most peripheral among candidate genes, trying to avoid compromising strain robustness. *YER134c*, *VBA5*, *YNR063w*, *YGR259c* have not previously been associated with phenotypes which disrupt important cell pathways.

Depending on the initial choice of perturbation, different subsets of deletions yielding the same outcome could likely be generated. When the corresponding null deletions do not cause a significant growth impediment the phenotypes observed in heterozygous background translate equally well or better in haploid deletions. An iterative carotenogenic screen could also be established in a haploid genetic background based on a Synthetic Genetic Array (SGA) approach [43], if additional selection markers are used to compile a set of four perturbations.

The availability of endogenous GGPP is a major limiting factor. Overexpression of the native BTS1 responsible for catalysing GGPP formation would most likely not be able to efficiently convert the FPP formed. It is likely that in *S. cerevisiae* only low levels of GGPP are required. Increased levels of the substrates FPP and GGPP of the mevalonate pathway cause feedback inhibition by destabilizing hmgp [41]. To minimize this effect, we chose to overexpress a variant of *ERG20* (F96C), capable of synthesizing all three steps leading to GGPP, as fusion to the 8OH-CPP synthase CLS [33,44]. Overexpression of CLS-*ERG20* (F96C) likely siphons GGPP towards 8OH-CPP and finally sclareol, preventing feedback inhibition. In the engineered strains expression of CLS-*ERG20* (F96C), excess byproduct FPP, seen as released NOH + FOH, is increasingly detectable. This could be caused by increased isoprene availability compared to wild type cells and possibly increased tolerance to higher FPP and GGPP levels. The ERAD complex plays an important role in controlling hmgp stability and effecting feedback inhibition from accumulating substrates. Surprisingly no mutants in the ERAD pathway were identified in the current screen. The stability of hmgp remained unaltered, unlike in previous series of strains developed optimized for sesquiterpenes. Enhanced formation of mevalonic acid could be due to transcriptional activation of HMGR rather than extended protein stabilization in this case. Still, overexpression of the catalytic domain of *HMG2* provided an additional boost, a bit higher than when *HMG2* (K6R) was overexpressed, unlike the case of previous hmgp stabilized strains, where only *HMG2* (K6R) exerted a positive effect. The excess FPP formed as side product of the *ERG20* (F96C) variant appeared in the new strains to follow unhindered the later pathway steps to ergosterol formation resulting in excess sterols formed. In the case, of the final AM238 strain co-expressing CLS-*ERG20* (F96C), CD-*HMG2* and sclareol synthase SCLS, the total amounts of released terpenol byproducts, which failed to be converted to sclareol, is lower than in wild type cells, indicating that part of the explanation is more efficient substrate utilization towards sclareol production. Nevertheless, the synthesis of higher amounts of squalene and ergosterols shows that additional improvements in substrate conversion towards diterpene production can be engineered.

## Conclusion

Aiming to increase diterpenoid production in yeast, we developed and applied an iterative yeast deletion screening approach based on carotenoid formation. The approach identifies series of deletions in tandem which are compatible between them, leading to enhanced carotenoid formation. An additional criterion selected for genes with minimal number of genetic and physical interactions. This enabled the identification of six gene deletions (*rox1, dos2, yer134c, vba5, ynr063w and ygr259c*) with no obvious growth impediments, while increasing 12-fold the yield of sclareol. Such approach could be applied in various selection schemes for which there is an easily scorable phenotype.

## Methods

### Chemicals and materials

Potassium hydroxide pellets, acetone (≥98%), ergosterol (≥98%) and squalene (≥98%) used as standards and petroleum ether (b.p. = 40–60°C) were all purchased from Sigma-Aldrich, while ethanol absolute was supplied from Scharlau.; Sclareol, kindly provided by VIORYL, SA, (−)-trans-caryophyllene (Sigma, C9653-5), were used as standard compounds; MyTaq DNA polymerase (BIO-21105, Bioline), and Accuzyme DNA polymerase (BIO-21051, Bioline) were used in PCR amplifications; NucleoSpin Plasmid Kit (REF 740588.250, Macherey-Nagel) was used for plasmid DNA purification; QIAquick Gel Extraction Kit (#28704, Qiagen) was used for gel extraction and DNA purification.

Yeast media: D (+)-Glucose monohydrate (16301, Sigma); Yeast Nitrogen Base w/o AA, carbohydrate & w/AS (Y2025, US Biologicals); Complete Minimal (CM) medium is composed of 0.13% (w/v) dropout powder (all essential amino acids) [45], 0.67% (w/v) yeast nitrogen base w/o AA, 2% glucose; TOPO TA Cloning Kit Dual Promoter (K4610-20, Invitrogen). All yeast transformations were done by lithium acetate transformation.

### Plasmids and yeast strain development

Explanations about the plasmids used can be found in Additional file 2: Table S2. All primer sequences used for strain development are listed in Table 3. The wild type yeast strain EG60 was transformed with the pUG27 cassette which contains the HIS5 selection marker flanked by loxP sites [46]. This was amplified with primers ROX1-pUG F and ROX1-pUG R primers, which incorporate flanking sequences complementary to the 5’ and 3’ of the *ROX1* gene respectively. Proper integration of the pUG27 cassette was verified by PCR from extracted genomic DNA using ROX1prom and ROX1 pUG R primers. The cassette was subsequently excised giving rise to strain AM228. The *DOS2* allele was deleted in AM228 cells using primers DOS2 Out pUG F and DOS2 OutR pUGR. Proper integration of the pUG27 cassette was verified by PCR from extracted genomic DNA using DOS2prom and DOS2 OutR pUG R primers. The cassette was subsequently excised giving rise to strain AM229. The *YER134c* allele was deleted in AM229 cells using primers YER134c pUG F and YER134c pUGR. Proper integration of the pUG27 cassette was verified by PCR from extracted genomic DNA using YER134c prom and YER134c pUG R primers. The cassette was subsequently excised giving rise to strain AM230. The *VBA5* allele was deleted in AM230 cells using primers VBA5-pUG F and VBA5-pUGR. Proper integration of the pUG27 cassette was verified by PCR from extracted genomic DNA using VBA5 prom and VBA5-pUG R primers. The cassette was subsequently excised giving rise to strain AM231. The *YNR063w* allele was deleted in AM231 cells using primers YNR063w-pUG F and YNR063w-pUGR. Proper integration of the pUG27 cassette was verified by PCR from extracted genomic DNA using YNR063w prom and YNR063w-pUGR primers. The cassette was subsequently excised giving rise to strain AM233. The *YGR259c* allele was deleted in AM233 cells using primers YGR259c-pUG F and YGR259c-pUGR. Proper integration of the pUG27 cassette was verified by PCR from extracted genomic DNA using YGR259c prom and YGR259c-pUGR primers. The cassette was subsequently excised giving rise to strain AM238.

**Table 3:** **List of primers used**

ROX1-pUG F primer	ttatacatttacggtgtcttaactctccctcttcacccctcattattccagaacagctgaagcttcgtacgc
ROX1-pUG R primer	caggagccaaatgcataaatttttagttaaagggaatatagtataatataatgcataggccactagtggatctg
ROX1prom	gctctatcttatttgctaattgtagtttc
DOS2 Out pUG F	gccttacatcaaatagcggtgattaatgataaaaagcacttagcagaagtcatgcacagctgaagcttcgtacgc
DOS2 OutR pUGR	atcacgcccagattttgtcttcctcctgtgcatctcttggattgatgattttcgcataggccactagtggatctg
DOS2prom	ctgggtattcatagcaattgtgaacccata
VBA5-pUG F	atgatacgagtcgacaaaatatgcaaaagataatagtgtcatcacacctttatgacagctgaagcttcgtacgc
VBA5-pUG R	gaaattacattccattgcgatacacctatttgattctgattgtgttgaagtctgtagcataggccactagtggatctg
VBA5prom	agtaggtgaaagttaacatgcgagt
YER134c pUG F	aagagaacaattttgttacaaatcctgctccaaaatagtctcaacgcgtttattcagctgaagcttcgtacgc
YER134c pUG R	tgacttcttcttatgctcagatgatgctctgtgaactaagtgcgcagtccttcagcataggccactagtggatctg
YER134c prom	tttcacgcactagaagaaggacct
YNR063w pUG F	ctgtgtctgttctctttgatgctgtcatctaagattcaattaaagtcgaccacagctgaagcttcgtacgc
YNR063w pUG R	ctaccatgtcatatggatctttcccctagtaaacttgtatctcaaaaggtcagcataggccactagtggatctg
YNR063w prom	ggtctattaggctctttactttgtaag
YGR259c pUG F	caggtcaaacagatactcatcattaatggcggacccataattttcagaaggttcagctgaagcttcgtacgc
YGR259c pUG R	tcttagaagtcgtattcacatcacagttttccccctcttcgcctttttcaaactagcataggccactagtggatctg
YGR259c prom	agcgaacctcagagcatattgttctc

### Construction of heterozygous deletion strains

Haploid cells *MATα* strains (EG60, AM228, AM229, AM230, AM231, AM233, AM238) carrying either episomally or integrated into the chromosome the YEplac195-YB/I/E plasmid were able to grown in the absence of uracil. They were mated out with *MATa* BY4741 library of deletion strains or a subset of the library and selected in glucose CM plates lacking uracil and tryptophan.

### Extraction and assay of carotenoids

Yeast cells growing on selective solid media were used to inoculate 50 mL liquid cultures with the corresponding media in 100 mL Erlenmeyer flasks which were then incubated for 5 days at 30°C. After cultivation, cells were collected by centrifugation (4000 rpm, 6.3 min) and the pellet was re-suspended with 1 mL de-ionized water. The cells were spun down (14680 rpm, 2 min) and dried in a speedvac under full vacuum conditions (30°C, 5 h). About 0.1 g of the dried cells was grounded, supplied with 2 mL dimethyl sulfoxide (DMSO), incubated with continuous vortexing at 55°C for 20 min, and finally sonicated for 10 min. The extracted carotenoids were collected and the cells were re-extracted by repeated vortexing with glass-beads and DMSO. A suitable amount of acetone: hexane: 20% NaCl (1:1:1 v/v) was added to the total supernatant extract to obtain the carotenoids into an organic phase. The mixture was vortexed for 1 min and centrifuged (4000 rpm, 6.3 min) to separate the two phases. The upper phase was then transferred into an amber glass vial and the organic solvent was evaporated under nitrogen purge at ambient temperature. The product was dissolved in hexane and the total carotenoid content was measured with spectrometry by recording its absorbance at 450 nm. The concentration of the carotenoids was calculated in accordance to [47] and expressed as μg/g dried cells.

### Sclareol analysis

5 ml cultures in the appropriate selection media were grown overnight at 30°C rotating in a roller drum. The next day 500 μl of dodecane (1:10 vol.) was added to each tube, and the cultures were incubated for two additional days. At the end of the incubation period the organic layer was removed into Eppendorf tubes, centrifuged to separate any residual medium left, and 200 μl were loaded on GC vials and analyzed by GC-MS for product identification and GC-FID for quantification.

### Sterol and squalene extraction

Yeast cells growing on selective plates media were used to inoculate 30 mL liquid cultures with the corresponding media in 100 mL Erlenmeyer flasks which were then incubated for 3 days at 30°C. After cultivation, cells were collected by centrifugation (4000 rpm, 6.3 min) and the pellet was re-suspended with 1 mL de-ionized water in order to be transferred into a pre-weighted 2 mL eppendorf tube. The cells were spun down (14680 rpm, 2 min) and weighted after the removal of the supernatant water. Subsequently, the pellet was mixed with a 16 mL EtOH/H_2_O 60% (v/v) mixture containing 25% KOH in a glass tube equipped with a plastic cap and saponified at 80 ± 2°C for 2 h. Non-saponificable sterols and squalene were extracted with 10 mL of petroleum ether, and the organic phase was collected dried under a N_2_ stream. The final extract was then re-suspended in 0.5 mL acetone and filtered through a PTFE syringe filter (0.45 μm x 25 mm) before GC analysis. For the qualitative and quantitative analysis, stock solutions of ergosterol and squalene in acetone were also made and calibration curves were drawn from the prepared working solutions. Yeast extracts were analyzed using a GC-2010 Plus Shimadzu gas chromatograph equipped with a flame ionization detector, and a MEGA-5MS capillary column (30 m x 0.25 mm, 0.25 μm film thickness), in the splitless mode. The temperature of injector and detector was 250°C and 320°C respectively. The oven temperature was initially held at 250°C, then increased slowly with a rate of 2°C/min up to 300°C and maintained at this temperature for 2 min to equilibrate. The temperature was subsequently raised with 1°C/min at 310°C and kept isothermally for 5 min in order to elute compounds with higher boiling points. The carrier gas used for the analysis was helium at a flow rate of 1.3 mL/min.

### Flow cytometric studies

The plasmid construct pUTDH3m/HMG1-TM-EYFP and was transformed to EG60, AM228, AM229, AM230, AM233, AM238 and AM109, AM133 cells. Overnight cultures of transformed cells were used to inoculate 50 ml cultures, and cells were grown to mid-log phase. 50,000 cells from each sample were measured for EYFP fluorescence by flow cytometry using the BD FACS calibur. For Nile red staining, five days old saturated cultures of EG60, AM228, AM229, AM230, AM233, AM238 cells carrying either empty vector or expressing CLS-ERG20 (F96C) were diluted to OD_600_ = 1 and stained for 10 min. with Nile red final concentration at 0.4 μg/ml. 50.000 cells were measured by flow cytometry, and mean fluorescence values were calculated. Overlay diagrams were done using the Flowing Software version 2.5.1. (http://www.flowingsoftware.com/).

## Additional files

Additional file 1: Table S1. List of gene deletions selected by the carotenoid screen. Additional file 2: Table S2. List of plasmids used in the study.

## References

[1] Vickers CE, Bongers M, Liu Q, Delatte T, Bouwmeester H (2014). Metabolic engineering of volatile isoprenoids in plants and microbes. Plant Cell Environ.

[2] Paddon CJ, Westfall PJ, Pitera DJ, Benjamin K, Fisher K, McPhee D (2013). High-level semi-synthetic production of the potent antimalarial artemisinin. Nature.

[3] WHO Malaria Policy Advisory Committee and Secretariat (2013). Malaria policy advisory committee to the WHO: conclusions and recommendations of september 2012 meeting. Malar J.

[4] Ro DK, Paradise EM, Ouellet M, Fisher KJ, Newman KL, Ndungu JM (2006). Production of the antimalarial drug precursor artemisinic acid in engineered yeast. Nature.

[5] Saloustros E, Mavroudis D, Georgoulias V (2008). Paclitaxel and docetaxel in the treatment of breast cancer. Expert Opin Pharmacother.

[6] Vasey PA (2008). Ovarian cancer: front-line standard treatment in 2008. Ann Oncol.

[7] Westfall PJ, Pitera DJ, Lenihan JR, Eng D, Woolard FX, Regentin R (2012). Production of amorphadiene in yeast, and its conversion to dihydroartemisinic acid, precursor to the antimalarial agent artemisinin. Proc Natl Acad Sci U S A.

[8] First international commercial flight completed with newly approved amyris-total aviation biofuel. https://amyris.com/first-international-commercial-flight-completed-with-newly-approved-amyris-total-aviation-biofuel/

[9] Ignea C, Pontini M, Maffei ME, Makris AM, Kampranis SC. Engineering monoterpene production in yeast using a synthetic dominant negative geranyl diphosphate synthase. ACS Synthetic Biol. 2014. doi:10.1021/sb400115e 10.1021/sb400115e 24847684

[10] Renninger NS, Ryder JA, Fisher KJS (2008). Jet fuel compositions and methods of making and using same.

[11] Peralta-Yahya PP, Ouellet M, Chan R, Mukhopadhyay A, Keasling JD, Lee TS (2011). Identification and microbial production of a terpene-based advanced biofuel. Nat Commun.

[12] Peralta-Yahya PP, Zhang F, del Cardayre SB, Keasling JD (2012). Microbial engineering for the production of advanced biofuels. Nature.

[13] Wriessnegger T, Augustin P, Engleder M, Leitner E, Muller M, Kaluzna I (2014). Production of the sesquiterpenoid (+)-nootkatone by metabolic engineering of Pichia pastoris. Metab Eng.

[14] Diaz-Chavez ML, Moniodis J, Madilao LL, Jancsik S, Keeling CI, Barbour EL (2013). Biosynthesis of Sandalwood Oil: Santalum album CYP76F cytochromes P450 produce santalols and bergamotol. PLoS One.

[15] Caniard A, Zerbe P, Legrand S, Cohade A, Valot N, Magnard JL (2012). Discovery and functional characterization of two diterpene synthases for sclareol biosynthesis in Salvia sclarea (L.) and their relevance for perfume manufacture. BMC Plant Biol.

[16] Schalk M, Pastore L, Mirata MA, Khim S, Schouwey M, Deguerry F (2012). Toward a biosynthetic route to sclareol and amber odorants. J Am Chem Soc.

[17] McGarvey DJ, Croteau R (1995). Terpenoid metabolism. Plant Cell.

[18] Jennewein S, Park H, DeJong JM, Long RM, Bollon AP, Croteau RB (2005). Coexpression in yeast of Taxus cytochrome P450 reductase with cytochrome P450 oxygenases involved in Taxol biosynthesis. Biotechnol Bioeng.

[19] Asadollahi MA, Maury J, Moller K, Nielsen KF, Schalk M, Clark A (2008). Production of plant sesquiterpenes in Saccharomyces cerevisiae: effect of ERG9 repression on sesquiterpene biosynthesis. Biotechnol Bioeng.

[20] Scalcinati G, Partow S, Siewers V, Schalk M, Daviet L, Nielsen J (2012). Combined metabolic engineering of precursor and co-factor supply to increase alpha-santalene production by Saccharomyces cerevisiae. Microb Cell Fact.

[21] Kampranis SC, Makris AM. Developing a yeast cell factory for the production of terpenoids. Comput Struct Biotechnol J. 2012;3:doi:10.5936/csbj.201210006. 10.5936/csbj.201210006 PMC3962098 24688666

[22] Polakowski T, Stahl U, Lang C (1998). Overexpression of a cytosolic hydroxymethylglutaryl-CoA reductase leads to squalene accumulation in yeast. Appl Microbiol Biotechnol.

[23] Hampton RY, Bhakta H (1997). Ubiquitin-mediated regulation of 3-hydroxy-3-methylglutaryl-CoA reductase. Proc Natl Acad Sci U S A.

[24] Ignea C, Cvetkovic I, Loupassaki S, Kefalas P, Johnson CB, Kampranis SC (2011). Improving yeast strains using recyclable integration cassettes, for the production of plant terpenoids. Microb Cell Fact.

[25] Babiskin AH, Smolke CD (2011). Synthetic RNA modules for fine-tuning gene expression levels in yeast by modulating RNase III activity. Nucleic Acids Res.

[26] Costanzo M, Baryshnikova A, Bellay J, Kim Y, Spear ED, Sevier CS (2010). The genetic landscape of a cell. Science.

[27] Ignea C, Trikka FA, Kourtzelis I, Argiriou A, Kanellis AK, Kampranis SC (2012). Positive genetic interactors of HMG2 identify a new set of genetic perturbations for improving sesquiterpene production in Saccharomyces cerevisiae. Microb Cell Fact.

[28] Alper H, Miyaoku K, Stephanopoulos G (2006). Characterization of lycopene-overproducing E. coli strains in high cell density fermentations. Appl Microbiol Biotechnol.

[29] Ozaydin B, Burd H, Lee TS, Keasling JD (2012). Carotenoid-based phenotypic screen of the yeast deletion collection reveals new genes with roles in isoprenoid production. Metab Eng..

[30] Reyes LH, Gomez JM, Kao KC (2014). Improving carotenoids production in yeast via adaptive laboratory evolution. Metab Eng.

[31] Ukibe K, Hashida K, Yoshida N, Takagi H (2009). Metabolic engineering of Saccharomyces cerevisiae for astaxanthin production and oxidative stress tolerance. Appl Environ Microbiol.

[32] Verwaal R, Wang J, Meijnen JP, Visser H, Sandmann G, van den Berg JA (2007). High-level production of beta-carotene in Saccharomyces cerevisiae by successive transformation with carotenogenic genes from Xanthophyllomyces dendrorhous. Appl Environ Microbiol.

[33] Ignea C, Trikka F., Nikolaidis A.K., Georgantea P., Ioannou E., Loupassaki S, et al. Efficient diterpene production in yeast by engineering Erg20p into a geranylgeranyl diphosphate synthase. 2014. In Press. 10.1016/j.ymben.2014.10.008 25446975

[34] Springer M, Weissman JS, Kirschner MW (2010). A general lack of compensation for gene dosage in yeast. Mol Syst Biol.

[35] Francois IE, Bink A, Vandercappellen J, Ayscough KR, Toulmay A, Schneiter R (2009). Membrane rafts are involved in intracellular miconazole accumulation in yeast cells. J Biol Chem.

[36] Blake JA, Dolan M, Drabkin H, Hill DP, Li N, Sitnikov D (2013). Gene Ontology annotations and resources. Nucleic Acids Res.

[37] Shimazu M, Itaya T, Pongcharoen P, Sekito T, Kawano-Kawada M, Kakinuma Y (2012). Vba5p, a novel plasma membrane protein involved in amino acid uptake and drug sensitivity in Saccharomyces cerevisiae. Biosci Biotechnol Biochem.

[38] Llorente B, Dujon B (2000). Transcriptional regulation of the Saccharomyces cerevisiae DAL5 gene family and identification of the high affinity nicotinic acid permease TNA1 (YGR260w). FEBS Lett.

[39] Ignea C, Trikka F, Nikolaidis AK, Georgantea P, Ioannou E, Loupassaki S (2015). Efficient diterpene production in yeast by engineering Erg20p into a geranylgeranyl diphosphate synthase. Metab Eng.

[40] Montanes FM, Pascual-Ahuir A, Proft M (2012). Repression of ergosterol biosynthesis is essential for stress resistance and is mediated by the Hog1 MAP kinase and the Mot3 and Rox1 transcription factors. Mol Microbiol.

[41] Garza RM, Tran PN, Hampton RY (2009). Geranylgeranyl pyrophosphate is a potent regulator of HRD-dependent 3-Hydroxy-3-methylglutaryl-CoA reductase degradation in yeast. J Biol Chem.

[42] Henry KW, Nickels JT, Edlind TD (2002). ROX1 and ERG regulation in Saccharomyces cerevisiae: implications for antifungal susceptibility. Eukaryot Cell.

[43] Tong AH, Boone C (2006). Synthetic genetic array analysis in Saccharomyces cerevisiae. Methods Mol Biol.

[44] Falara V, Pichersky E, Kanellis AK (2010). A copal-8-ol diphosphate synthase from the angiosperm Cistus creticus subsp. creticus is a putative key enzyme for the formation of pharmacologically active, oxygen-containing labdane-type diterpenes. Plant Physiol.

[45] Treco D, Lundblad V (1993). Basic Techniques of yeast genetics. Current protocols in molecular biology.

[46] Gueldener U, Heinisch J, Koehler GJ, Voss D, Hegemann JH (2002). A second set of loxP marker cassettes for Cre-mediated multiple gene knockouts in budding yeast. Nucleic Acids Res.

[47] Scott KJ. Detection and Measurement of Carotenoids by UV/VIS Spectrophotometry. In: Current Protocols in Food Analytical Chemistry. John Wiley & Sons, Inc.; 2001. http://dx.doi.org/10.1002/0471142913.faf0202s00

