# Supplementary material for: Iterative carotenogenic screens identify combinations of yeast gene deletions that enhance sclareol production
Source: Microb Cell Fact. 2015 Apr 24;14:60. doi: 10.1186/s12934-015-0246-0 (PMC4413541; doi:10.1186/s12934-015-0246-0)
Supplement: Additional file 1: Table S1. — List of gene deletions selected by the carotenoid screen. [file 12934_2015_246_MOESM1_ESM.doc]

| Gene name | ORF | Description | Biological process |
| --- | --- | --- | --- |
| *ADE3* | YGR2014w | Cytoplasmic trifunctional enzyme C1-tetrahydrofolate synthase | Purine nucleobase biosynthetic process |
| *AIM19* | YIL087C | Putative protein of unknown function; the authentic, non-tagged protein is detected in purified mitochondria in high-throughput studies; null mutant displays reduced respiratory growth | Uknown |
| *AIM22* | YJL046W | Putative lipoate-protein ligase; required along with Lip2 and Lip5 for lipoylation of Lat1p and Kgd2p | Protein lipoylation |
| *ALK1* | YGL021W | Protein kinase; required for proper spindle positioning and nuclear segregation following mitotic arrest, proper organization of cell polarity factors in mitosis, | Protein phosphorylation; mitotic nuclear division |
| *ATG2* | YNL242W | Peripheral membrane protein required for autophagic vesicle formation; also required for vesicle formation during pexophagy and the cytoplasm-to-vaucole targeting (Cvt) pathway | Membrane; organelle assembly; peroxisome organisation; vacuole organisation |
| *ATG27* | YJL178C | Type I membrane protein involved in autophagy and the Cvt pathway; may be involved in membrane delivery to the phagophore assembly site | Membrane; organelle assembly; peroxisome organization; vacuole organization |
| *ATP10* | YLR393W | Assembly factor for the F0 sector of mitochondrial F1F0 ATP synthase | Mitochondrial proton transporting ATP-synthase complex assembly |
| *BCP1* | YDR361C | Essential protein involved in nuclear export of Mss4p | Ribosomal subunit export from nucleus |
| *BER1* | YLR412W | Protein involved in microtubule-related processes | Microtubule-based process |
| *BLS1* | YLR408C | Subunit of the BLOC-1 complex involved in endosomal maturation | Endosome organization; regulation of protein localization |
| *BNA3* | YJL060W | Kynurenine aminotransferase | Kynurenic acid biosynthetic process |
| *CDC40* | YDR364C | Pre-mRNA splicing factor; important for catalytic step II of pre-mRNA splicing and plays a role in cell cycle progression | Cell cycle G1/S phase transition; mRNA 3'-splice site recognition; generation of catalytic spliceosome for second transesterification step |
| *CHS5* | YLR330W | Component of the exomer complex | Conjugation; site of polarized growth |
| *CIR2* | YOR356W | Putative ortholog of human ETF-dH; found in a large supramolecular complex with other mitochondrial dehydrogenases |  |
| *CKB1* | YGL019W | Beta regulatory subunit of casein kinase 2 (CK2) | Protein phosphorylation; regulation of transcription from PolI and PolIII promoter; cell response to DNA damage |
| *CLG1* | YGL215W | Cyclin-like protein that interacts with Pho85p | Regulation of cell cycle; positive regulation of macroautophagy |
| *COQ4* | YDR204W | Protein with a role in ubiquinone (Coenzyme Q) biosynthesis | Ubiquinone biosynthetic process |
| *COX19* | YLL018C-A | Protein required for cytochrome c oxidase assembly | Metal ion transport; mitochondrial respiratory chain assembly |
| *CPR1* | YDR155C | Cytoplasmic peptidyl-prolyl cis-trans isomerase (cyclophilin) | Histone modification |
| *CSF1* | YLR087C | Protein required for fermentation at low temperature | Fermentation; protein maturation |
| *CUE5* | YOR042W | Ubiquitin-binding protein; functions as a ubiquitin-Atg8p adaptor in ubiquitin-dependent autophagy | Small conjugating protein binding |
| *CWH41* | YGL027C | Processing alpha glucosidase I | Hydrolase activity; protein glycosylation |
| *CYK3* | YDL117W | SH3-domain protein located in the bud neck and cytokinetic actin ring | Site of polarised growth |
| *DFG16* | YOR030W | Probable multiple transmembrane protein; involved in diploid invasive and pseudohyphal growth upon nitrogen starvation | Membrane; protein maturation |
| *DLD2* | YDL178W | D-lactate dehydrogenase | Cytoskeletal protein binding; monocarboxylic acid metabolic processing; oxidoreductase activity |
| *DON1* | YDR273W | Meiosis-specific component of the spindle pole body; paralog of CUE5 | Membrane; sporulation |
| *DOS2* | YDR068W | Protein of unknown function |  |
| *DUG1* | YFR044C | Cys-Gly metallo-di-peptidase | Hydrolase activity |
| *ERG3* | YLR056W | C-5 sterol desaturase | Lipid metabolic process |
| *ERV15* | YBR210W | Protein involved in export of proteins from the endoplasmic reticulum | Cell budding; Golgi vesicle transport |
| *EST2* | YLR318W | Reverse transcriptase subunit of the telomerase holoenzyme | DNA replication |
| *EXG1* | YLR300W | Major exo-1,3-beta-glucanase of the cell wall | Cell wall; hydrolase activity, acting on glycosyl bonds |
| *FAT3* | YKL187C | Protein required for fatty acid uptake | Long-chain fatty acid transport |
| *FBP26* | YJL155C | Fructose-2,6-bisphosphatase | Glucose metabolic process |
| *HMF1* | YER057C | Member of the p14.5 protein family |  |
| *HSP78* | YDR258C | Oligomeric mitochondrial matrix chaperone | Mitochondrial organization; protein folding; response to heat |
| *ICT1* | YLR099C | Lysophosphatidic acid acyltransferase | Transferase activity |
| *IKS1* | YJL057C | Protein kinase of unknown cellular role | Kinase activity; transferase activity |
| *IME1* | YJR094C | Master regulator of meiosis that is active only during meiotic events | Protein binding transcription factor activity; sporulation |
| *IMH1* | YLR309C | Protein involved in vesicular transport | Protein targeting |
| *IPT1* | YDR072C | Inositolphosphotransferase; involved in synthesis of mannose-(inositol-P)2-ceramide (M(IP)2C) | Transferase activity |
| *ITR2* | YOL103W | Myo-inositol transporter | Membrane; transmembrane transporter activity |
| *LCL1* | YPL056C | Putative protein of unknown function; deletion mutant is fluconazole resistant and has long chronological lifespan | Membrane |
| *LDB7* | YBL006C | Component of the RSC chromatin remodeling complex | Chromatin organization; DNA-templated transcription; elongation |
| *LEA1* | YPL213W | Component of U2 snRNP complex | mRNA processing |
| *LOT6* | YLR011W | FMN-dependent NAD(P)H:quinone reductase; role in apoptosis-like cell death | Response to oxidative stress |
| *MBA1* | YBR185C | Membrane-associated mitochondrial ribosome receptor | Protein complex biogenesis |
| *MEF1* | YLR069C | Mitochondrial elongation factor involved in translational elongation | Mitochondrial organization; RNA binding; translation factor; nucleic acid binding; translational elongation |
| *MEI4* | YER044C-A | Meiosis-specific protein involved in forming DSBs | Sporulation |
| *MNL2* | YLR057W | Putative mannosidase involved in ER-associated protein degradation | Hydrolase activity |
| *MRPL24* | YMR193W | Mitochondrial ribosomal protein of the large subunit | Mitochondrion organization; structural molecule activity |
| *MTC2* | YKL098W | Protein of unknown function |  |
| *NPR3* | YHL023C | Subunit of SEA (Seh1-associated),Npr2/3 complex mediates downregulation of TORC1 activity | Pseudohyphal growth; meiotic nuclear division, negative regulation of TOR signaling, regulation of autophagic assembly |
| *NTC20* | YBR188C | Member of the NineTeen Complex (NTC) | mRNA processing |
| *OST3* | YOR085W | Gamma subunit of the oligosaccharyltransferase complex of the ER lumen | Protein glycosylation; transferase activity |
| *PEP1* | YBL017C | Type I transmembrane sorting receptor for multiple vacuolar hydrolases | Protein targeting |
| *PGM1* | YKL127W | Phosphoglucomutase, minor isoform | Glycogen, Trehalose , UDP-glucose biosynthetic process; Glucose-1 phosphate, glucose-6 phosphate metabolic process; galactose catabolic process |
| *PLB3* | YOL011W | Phospholipase B (lysophospholipase) involved in lipid metabolism | Lipid metabolic process; membrane |
| *PTH1* | YHR189W | One of two mitochondrially-localized peptidyl-tRNA hydrolases | Mitochondrion organisation |
| *ROD1* | YOR018W | Alpha-arrestin involved in ubiquitin-dependent endocytosis | Endocytosis; membrane; response to chemical |
| *RPL24a* | YGL031C | Ribosomal 60S subunit protein L24A; not essential for translation but may be required for normal translation rate | Structural molecule activity |
| *RVS167* | YDR388W | Actin-associated protein with roles in endocytosis and exocytosis | Cytoskeletal protein binding; site of polarized growth |
| *SEC28* | YIL076W | Epsilon-COP subunit of the coatomer; regulates retrograde Golgi-to-ER protein traffic | Golgi vesicle transport; vesicle organisation |
| *SEC72* | YLR292C | Non-essential subunit of Sec63 complex | Posttranslational protein targeting to membrane; translocation |
| *SFK1* | YKL051W | Plasma membrane protein that may act to generate normal levels of PI4P | Cytoskeleton organisation; membrane |
| *SHM2* | YLR058C | Cytosolic serine hydroxymethyltransferase | Membrane; site of polarised growth |
| *SIR3* | YLR442C | Silencing protein | Chromatin organisation; histone binding |
| *SKN1* | YGR143W | Protein involved in sphingolipid biosynthesis | 1 -6 beta-D-glucan biosynthesis. Pr; sphingolipid biosynthesis. Pr.; fungal type cell wall organization |
| *SLT2* | YHR030C | Serine/threonine MAP kinase; involved in regulating maintenance of cell wall integrity, cell cycle progression | Kinase activity; mRNA processing; peroxisome organization; site of polarized growth; transferase activity |
| *SNX3* | YOR357C | Sorting nexin for late-Golgi enzymes | Golgi vesicle transport |
| *STM1* | YLR150W | Protein required for optimal translation under nutrient stress; perturbs association of Yef3p with ribosomes; involved in TOR signaling | Telomere organisation |
| *SUR1* | YPL057C | Mannosylinositol phosphorylceramide (MIPC) synthase catalytic subunit | Transferase activity |
| *SUR4* | YLR372W | Elongase; involved in fatty acid and sphingolipid biosynthesis | Golgi vesicle transport; monocarboxylic acid metabolic process |
| *SWA2* | YDR320C | Auxilin-like protein involved in vesicular transport | Organelle inheritance; small conjugating protein binding |
| *SYM1* | YLR251W | Protein required for ethanol metabolism homologous to mammalian peroxisomal membrane protein Mpv17 | Ethanol metabolic process |
| *TIF2* | YJL138C | Translation initiation factor eIF4A | Membrane; regulation of translation; RNA binding; translation factor activity; nucleic acid binding |
| *TPO3* | YPR156C | Polyamine transporter of the major facilitator superfamily | Spermine transport |
| *TYW1* | YPL207W | Iron-sulfer protein required for synthesis of Wybutosine modified tRNA | Wybutosine biosynthetic process |
| *VBA5* | YKR105C | Plasma membrane protein of the Major Facilitator Superfamily (MFS); involved in amino acid uptake and drug sensitivity | Membrane |
|  | YBL028C | Protein of unknown function that may interact with ribosomes |  |
|  | YBL029W | ORF, uncharacterised |  |
|  | YDR114c | Putative protein of unknown function; deletion mutant exhibits poor growth at elevated pH and calcium |  |
|  | YDR262w | Putative protein of unknown function; green fluorescent protein (GFP)-fusion protein localizes to the vacuole and is induced in response to the DNA-damaging agent MMS |  |
| *YEH2* | YLR020C | Steryl ester hydrolase; catalyzes steryl ester hydrolysis at the plasma membrane | Lipid metabolic process; membrane |
|  | YEL045C | Dubious open reading frame; deletion gives MMS sensitivity |  |
|  | YER134C | Magnesium-dependent acid phosphatase; member of the haloacid dehalogenase superfamily | Phosphatase activity; protein dephosphorylation |
| *YET1* | YKL065C | Endoplasmic reticulum transmembrane protein; may interact with ribosomes |  |
|  | YGR259C | Dubious open reading frame; ; overlaps almost completely with the verified ORF TNA1 |  |
| *BMT5* | YIL096C | Methyltransferase required for m3U2634 methylation of the 25S rRNA | Methyl transferase activity |
|  | YKL053W | Dubious open reading frame |  |
|  | YKL096C-B | Putative protein of unknown function |  |
|  | YNR063W | Putative zinc-cluster protein of unknown function | DNA binding |
|  | YNR073C | Putative mannitol dehydrogenase |  |
|  | YOL046C | Dubious open reading frame |  |
|  | YOR029C | Dubious open reading frame |  |
|  | YOR292C | Putative protein of unknown function; green fluorescent protein (GFP)-fusion protein localizes to the vacuole |  |
|  | YOR318C | Dubious open reading frame |  |
|  | YOR376W | Dubious open reading frame |  |
|  | YPR109W | Predicted membrane protein |  |
| *YVC1* | YOR087W | Vacuolar cation channel | Cellular ion homeostasis |
