# Supplementary material for: Iterative carotenogenic screens identify combinations of yeast gene deletions that enhance sclareol production
Source: Microb Cell Fact. 2015 Apr 24;14:60. doi: 10.1186/s12934-015-0246-0 (PMC4413541; doi:10.1186/s12934-015-0246-0)
Supplement: Additional file 2: Table S2. — List of plasmids used in the study. [file 12934_2015_246_MOESM2_ESM.pdf]

Plasmid name	Description	Explanation
YEplac195-YB/I/E	P_{TDH3}-crtYB-tCYC1, P_{TDH3}-crtI-tCYC1, P_{TDH3}-crtE-tCYC1, URA3, 2μ	Plasmid expressing GGPP synthase (crtE), phytoene/lycopene synthase (crtYB) and phytoene desaturase (crtI) genes from *X. dendrorhous*
pUTDH/CLS-ERG20 (F96C)	P_{TDH3}-*CcCLS-ERG20 (F96C)*-tCYC1, 2μ, *URA3*	Plasmid expressing a fusion between the Cistus creticus 8-hydroxy-copalyl diphosphate synthase (*CcCLS*) to a mutant form of *ERG20* (F96C)
pHTDH/CD-*HMG2*	P_{TDH3}-CD-*HMG2*-tCYC1, 2μ, *HIS3*	Plasmid expressing the catalytic domain of *HMG2*
*p*WTDH/*SCLSmat*	P_{TDH3}-*SCLS-tCYC1, 2μ TRP1*	Plasmid expressing the mature form of sclareol synthase (SCLS)
pUTDH3/*HMG1*-TM-EYFP	2μ P_{TDH3}-*HMG1*-TM-EYFP *URA3*	Plasmid expressing the transmembrane domain of *HMG1* fused to EYFP
pUTDH3/Sf126-EYFP	2μ P_{TDH3}-Sf126-EYFP *URA3*	Plasmid expressing caryophyllene synthase from *S. fruticosa* fused to EYFP
